# Mitochondrial proteome of the apicomplexan *Babesia divergens*

**DOI:** 10.1016/j.cub.2026.08.072

**Published:** 2026-10-01

**Authors:** Cora N. Betsinger, Sarah E. Calvo, Syed Yusuf Mian, Niel C. Bauer, Aditya S. Paul, Donovan L. Batzli, Cameron Genxuan Lian, Brian W. Boyle, Keith D. Rivera, Sreelakshmi K. Sreenivasamurthy, Jason G. McCoy, Michael Z. Chen, Tiana M. Issa, Jonathan A. Stefely, MitoCarta Tree of Life Consortium, Jeffrey D. Dvorin, Namrata D. Udeshi, Manoj T. Duraisingh, Vamsi K. Mootha

**Affiliations:** 1 Department of Molecular Biology, Massachusetts General Hospital, Boston, MA 02114, USA; 2 Harvard Medical School, Boston, MA 02115, USA; 3 Broad Institute of MIT and Harvard, Cambridge, MA 02142, USA; 4 Howard Hughes Medical Institute, Massachusetts General Hospital, Boston, MA 02114, USA; 5 Department of Immunology and Infectious Diseases, Harvard T.H. Chan School of Public Health, Boston, MA 02115, USA; 6 Division of Infectious Diseases, Boston Children’s Hospital, Boston, MA 02115, USA; 7 Department of Pediatrics, Harvard Medical School, Boston, MA 02115, USA

**Keywords:** Mitochondria, Apicomplexa, *Babesia*, *Plasmodium*, *Toxoplasma*, proteome, isocitrate dehydrogenase, electron transport chain, ATP synthase, mitochondrial ribosome

## Abstract

*Babesia* are tick-transmitted apicomplexan parasites of widespread medical and veterinary importance. Although atovaquone, a first-line therapy for human babesiosis, targets one of the three proteins encoded by mitochondrial DNA, a full characterization of the nuclear-encoded mitochondrial proteome is lacking. Here, we combined organelle immunoprecipitation and density gradient separation to enrich mitochondria from *Babesia divergens.* Using protein mass spectrometry and protein correlation profiling, we generated BdMitoCarta, a high confidence inventory of 525 mitochondrial proteins. The *B. divergens* mitoproteome is broadly conserved across Apicomplexa, which as a clade have undergone reductive evolution. As seen in other apicomplexans, metabolite transport and the TCA cycle have diverged, with loss of the pyruvate dehydrogenase complex, presence of malate:quinone oxidoreductase and a type I fumarase. Specific to *Babesia* is the presence of two mitochondrial isocitrate dehydrogenase homologs. Using isolated mitochondria, we performed Blue Native PAGE complexome analysis and detected previously uncharacterized proteins co-migrating with the ATP synthase and mitochondrial ribosome. Our work has yielded the first mitochondrial proteome and complexome for *Babesia*, which we expect to be a valuable resource for understanding the evolution of apicomplexan parasites for future therapeutic development.

## IN BRIEF

Betsinger and Calvo et al. characterize the mitochondrial proteome and complexome of *Babesia divergens,* a tick-borne parasite evolutionarily related to *Plasmodium* and *Toxoplasma*.

## Graphical Abstract

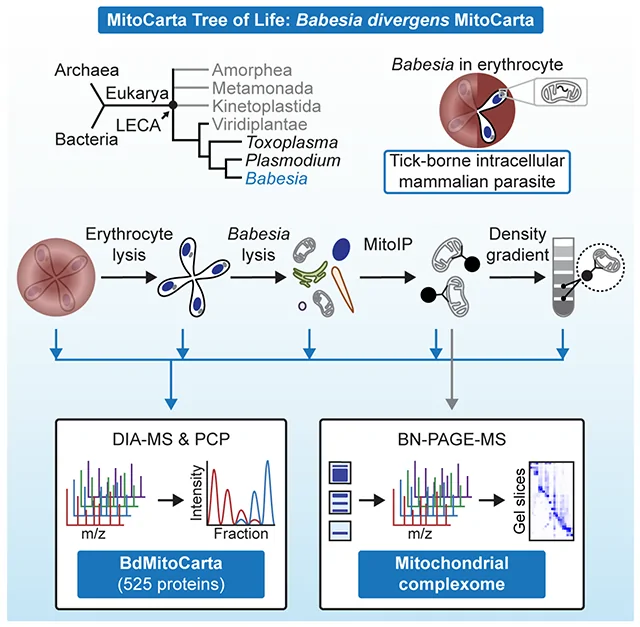

## INTRODUCTION

Mitochondria have been shaped by over one billion years of eukaryotic evolution.^1^ During this time, organisms have adapted these organelles to their needs by losing, gaining, and re-localizing mitochondrial proteins. This remodeling is especially pronounced in Apicomplexa, a phylum of primarily intracellular parasites that contain a relic plastid organelle called the apicoplast.^2,3^ Apicomplexa is part of the larger Alveolate group, which includes dinoflagellates and ciliates (Figure 1A). Together with Rhizaria and Stramenopiles, Alveolates make up the SAR supergroup, which is estimated to account for half of all eukaryotic diversity.^4^

Medically relevant apicomplexans include *Cryptosporidium, Toxoplasma, Plasmodium,* and *Babesia* (Figure 1A). These organisms tend to exhibit divergent, streamlined, and genera-specific mitochondrial biology that reflects their ability to scavenge metabolites from a parasitized host.^5–8^ Some apicomplexans such as *Cryptosporidium* have reduced their mitochondrion to a remnant organelle that has lost its mitochondrial genome (mtDNA), electron transport chain (ETC), and TCA cycle.^9,10^ In contrast, *Toxoplasma* and *Plasmodium* possess a small mtDNA that encodes three ETC subunits and continue to support aerobic ATP production.^5–8,11^ The existence of a plastid remnant organelle, the apicoplast, has also driven mitochondrial remodeling in apicomplexans, with loss of some mitochondria-localized processes in favor of analogous apicoplast pathways.^12–15^

Proteomic analysis of apicomplexan mitochondria can provide valuable mechanistic and evolutionary insights, but achieving high experimental accuracy is technically challenging. Apicomplexan organisms contain a single mitochondrion, which can be tethered to the apicoplast.^16,17^ Moreover, these protists are obligate intracellular parasites,^2,3^ and isolating mitochondria for mass spectrometry analysis requires host cell lysis followed in sequence with parasite lysis. Hence, obtaining a high yield of purified and intact mitochondria from these organisms is technically challenging. Bioinformatic approaches to proteomic characterization are limited by high sequence divergence, highlighting the importance of experimentally defined inventories.

Despite these challenges, there has been progress in defining mitochondrial proteins and proteomes from apicomplexan organisms. Subcellular proteomes, including of the mitochondrion, have been experimentally determined for *Toxoplasma gondii, Plasmodium falciparum,* and *Cryptosporidium parvum* using the hyperplexed localization of organelle proteins by isotope tagging (hyperLOPIT) method.^10,11,18^ A bioinformatics-based study employed a naïve Bayes strategy to integrate eight different data sets to computationally predict a list of 445 putative *P. falciparum* mitochondrial proteins, relying most heavily on homology to the *T. gondii* hyperLOPIT study, and generated the most comprehensive list for *Plasmodium*.^19^ Using proximity biotinylation, 122 *P. falciparum* and 421 *T. gondii* mitochondrial proteins were identified.^20,21^ Complexome profiling has helped to reveal an inventory of *P. falciparum* and *T. gondii* OXPHOS subunits.^22,23^

At present, the mitochondrial proteomes of *Babesia* species have yet to be investigated. The *Babesia* genus is comprised of hundreds of species distributed around the globe, with medical, veterinary, and agricultural importance.^24–27^ *Babesia* spends part of its lifecycle replicating within the erythrocytes of a vertebrate host, and half of its lifecycle replicating and developing within a tick host.^26,28^ *B. bovis, B. bigemina,* and *B. divergens* are agriculturally important species that cause bovine babesiosis, which annually results in significant global livestock loss.^29^ Several *Babesia* species are known to cause human babesiosis, including *B. divergens, B. microti, B. duncani,* and *B. venatorum.*^24,25,27,30^ While frequently asymptomatic, infection with *Babesia* can result in a malaria-like disease characterized by fevers and hemolytic anemia, which can be life-threatening. Due to climate-induced increases in tick ranges and human encroachment into tick habitats, cases of human babesiosis are becoming more common and geographically widespread.^30^

The mitochondrion is an established therapeutic target for treating human babesiosis, which is currently treated with a first-line therapy combination of atovaquone and azithromycin.^31,32^ Atovaquone acts by directly inhibiting the mtDNA-encoded CYTB protein of the ETC. Despite this clinical relevance, little is known about *Babesia* mitochondrial biology. Early genomic characterization revealed a highly reduced mtDNA of about 6 kilobases that only encodes three ETC proteins and fragmented ribosomal RNA genes.^8,33^ The limited information that we do have is derived from comparative genomic analyses with their distant evolutionary cousins *T. gondii* and *Plasmodium*.^34,35^

Here, as part of the MitoCarta Tree of Life Consortium (MitoTOL), we sought to characterize the mitochondrial proteome of *B. divergens.* Through extensive optimization, we developed a protocol that combines release of *B. divergens* from the host erythrocyte, parasite lysis, and application of affinity purification and density centrifugation to generate progressively enriched fractions of mitochondria. We then applied protein mass spectrometry proteomics followed by protein correlation profiling to generate the BdMitoCarta inventory of 525 mitochondrial proteins, which we estimate is the most comprehensive apicomplexan mitochondrial inventory to date. We also performed BN-PAGE complexome analysis of these mitochondria, which revealed *B. divergens* proteins co-migrating with the ATP synthase and mitoribosome.

## RESULTS

### An optimized experimental workflow to enrich mitochondria from *B. divergens*

To enrich intact mitochondria from *B. divergens* parasites for downstream proteomic analysis, we optimized host erythrocyte lysis, lysis of the isolated parasite cell, and subsequent separation of mitochondria from other organelles. To facilitate mitochondrial enrichment via immunoprecipitation (MitoIP), we generated a *B. divergens* recombinant line expressing 3xHA-eGFP-BdTOM22. TOM22 is a core, conserved subunit of the mitochondrial outer membrane (MOM) protein import complex.^36^ TOM22 has a single transmembrane segment with an amino terminus facing the cytosol.^36,37^ Thus, fusion of the 3xHA-eGFP tag to the amino terminus of BdTOM22 positions the affinity tags on the cytosolic side of the membrane (Figure S1A). We confirmed localization of the affinity handle to the mitochondrion in *B. divergens* merozoites both with expansion and immunofluorescence microscopy (Figure 1B, Figure S1B).

We cultured 3xHA-eGFP-BdTOM22 parasites to high parasitemia (~20–30% of erythrocytes infected). For erythrocyte lysis, we tested mechanical filtration, hypotonic lysis, detergent-based lysis (saponin and digitonin), and pore-forming toxins (streptolysin O). Mechanical filtration was the only method that resulted in robust erythrocyte lysis and parasite evacuation from the host cell membrane (Figure 1C). Free parasites were separated from erythrocyte contaminants by differential centrifugation. To disrupt the parasite plasma membrane and release intact organelles into solution, we used nitrogen cavitation. We then treated the organelle suspension with Benzonase nuclease to digest free DNA prior to incubating the organelles with magnetic anti-GFP beads for MitoIP.^38^ Affinity captured mitochondria were further purified by density gradient centrifugation. The mitochondria were first pelleted onto an OptiPrep cushion prior to floating them through a discontinuous OptiPrep step gradient and collecting the resulting bands.

Western blot analysis revealed progressive mitochondrial enrichment through the protocol (Figure 1D). Additionally, the mitochondrial outer membrane, intermembrane space, and matrix markers trended similarly, indicating the organelles remained intact. The whole cell lysate, crude mitochondria, MitoIP, and gradient band samples were further analyzed by mass spectrometry using data-independent acquisition (DIA) methods (Figure S2A).^39^ In total, mass spectrometry detected 3,410 protein groups (3,414 proteins) of the 4,132 annotated *B. divergens* proteins (Data S2). We note that this 82% detection is extremely high as *B. divergens* is not expected to express its entire proteome during blood stage development.^40^

To determine whether mitochondrial proteins were differentially enriched by our protocol, we used annotations transferred from the *T. gondii* subcellular proteomic dataset.^11^ We mapped homologs based on BLASTP bidirectional best hits (BBH). The 288 *B. divergens* proteins with BBH to mitochondrial proteins in *T. gondii* showed clear enrichment across the protocol, increasing from 7% of the total proteomic signal in whole cells, to 25% after MitoIP, to 39% after density gradient separation (Figure 1E). The putative mitochondrial proteins increased in relative abundance across the protocol and were equally enriched in gradient bands B1 and B2, whereas proteins belonging to other organelles, including the ER, apicoplast, and plasma membrane, were enriched only in the lighter gradient band B1 (Figure 1F). Therefore, while the purest sample still contained many non-mitochondrial proteins, there was a clear signature of variation across all samples that distinguished mitochondrial proteins from all other compartments.

### The *B. divergens* mitoproteome contains 525 proteins

To experimentally define the *B. divergens* mitochondrial proteome, we used mass spectrometrybased protein correlation profiling (PCP) (Data S3).^41^ This method is based on correlating protein abundance profiles from successively enriched mitochondrial fractions to profiles of known mitochondrial proteins. We selected TOM40, HSP60, and ISCU as the universal marker proteins for the MitoTOL consortium due to their high level of conservation across eukaryotic organisms.^42–44^ Additionally, these three proteins span the mitochondrial compartments from mitochondrial outer membrane to matrix. We note that since most benchmark mitochondrial proteins show similar profiles (Figure S2C), our method is robust to the exact markers selected. Of the 3,414 *B. divergens* proteins detected and quantified by mass spectrometry, our PCP analysis identified 520 proteins as mitochondrial, using a >0.96 correlation, selected as the threshold that maximizes area under the sensitivity-precision curve (Figure 1G). These 520 proteins make up our “MitoViaPCP” list. We note that the three mtDNA-encoded proteins (cox1, cox3, and cob), which are notoriously difficult to detect by mass spectrometry due to their small size and hydrophobicity, are all detected and present in the MitoViaPCP dataset, underscoring the sensitivity of our entire experimental approach. Moreover, this result is important since it indicates these three mtDNA genes continue to produce translated protein products.

The MitoViaPCP dataset has an estimated sensitivity of 83% and corrected false discovery rate (cFDR) of 13% based on benchmark datasets (Figure 1H, Data S4). The mitochondrial benchmark proteins were curated based on BBH homology to *P. falciparum* proteins with strong evidence of mitochondrial localization. Of the 82 benchmark proteins, 68/82 (83%) are in MitoViaPCP, 1/82 was not detected by mass spectrometry, and 13/82 proteins failed to meet the 0.96 correlation level threshold and may include dual localized proteins or species-specific differences.

To validate the robustness of the experimental workflow and analysis pipeline, we performed a biological replicate (Figure S2). The resulting MitoViaPCP dataset showed 95% overlap with the first replicate (Figure 2A, Figure S2F). Only 31 proteins not already in MitoViaPCP met the same PCP threshold in the second replicate, including five with corroborating evidence from *T. gondii* hyperLOPIT.^11^ We chose to define the *B. divergens* MitoCarta (BdMitoCarta) catalog as the union of 520 MitoViaPCP proteins and these five additional proteins (LplA/Bdiv_005150, COA4/Bdiv_037220c, LYRM4/Bdiv_027170c, Bdiv_032330, Pet191/Bdiv_030730), totaling 525 mitochondrial proteins (Figure 2A, Data S3).

The BdMitoCarta inventory is freely available online at mitocarta.org. To make the inventory more useful to the wider apicomplexan research community, we provide tables mapping these genes to *P. falciparum* and *T. gondii* identifiers (Data S3).

### Microscopy analysis of select BdMitoCarta proteins

We selected two categories of proteins for microscopy analysis: 5 proteins from the BdMitoCarta inventory and 4 proteins from our literature-based benchmark mitochondrial protein (“Gold+”) inventory that did not score highly in our MitoViaPCP analysis (Figure 2B). Of the five BdMitoCarta proteins, three (LYRM4, BdIDHL, and Bd_016970) showed mitochondrial localization in tetrads (Figure 2C) as well as in single and paired forms (Figure S3) and one (BdIDH) showed dual localization with mitochondria and another compartment. One of the tested BdMitoCarta proteins (LplA) failed to show mitochondrial localization; however, epitope tags can disrupt mitochondrial localization in some cases. Of the four low-scoring proteins in PCP analysis that were in our benchmark Gold+ dataset (EngA, FtsH, KsgA1, and TopoIII), we found that none showed convincing mitochondrial localization in *B. divergens* (Figure 2D). These discrepancies indicate either divergence in mitochondrial localization between *Babesia* and *Plasmodium*, or potential errors in the previous annotations.

### BdMitoCarta proteins are conserved in Apicomplexa

The 525 BdMitoCarta proteins mapped to 471 orthogroups (Chen et al, co-submitted), which were used to identify homologs in other apicomplexan species (Figure 3, Data S5). Note that in this analysis, we are evaluating whether BdMitoCarta homologs are present in other organisms, not necessarily in their mitochondria. Using these criteria, we find BdMitoCarta protein repertoires are largely conserved in *B. bigemina* (98% conservation) and *B. microti* (90% conservation), representative species of the *Babesia sensu stricto* and *sensu lato* groups, respectively. The BdMitoCarta proteome further shares extensive homologs with pathogenic *Plasmodium* and *T. gondii* species (>87% conservation) (Figure 3A). While mitochondrial proteins generally showed greater conservation than the background proteome rate, this trend was flipped in apicomplexan species that have lost their mtDNA (e.g. *Cryptosporidium* and *Gregarina*) (Figure 3A).

Given the conservation of BdMitoCarta with *Plasmodium knowlesi* (91% conservation), we integrated BdMitoCarta with the recent *P. knowlesi* essentialome dataset^45^ to predict the essentiality of the *B. divergens* mitoproteome. We observed that 253/471 (54%) BdMitoCarta orthogroups are essential in *P. knowlesi*, which is comparable to the essentiality of mitoproteome genes in *P. knowlesi*, in contrast to the *P. knowlesi* background rate of 39% (Figure 3B).

Next, we sought to compare our BdMitoCarta proteome (n = 525) to published hyperLOPIT studies^11,18^ that identified mitochondrial proteins in *P. falciparum* (n = 153) and *T. gondii* (n = 469) (Figure S4, Data S5C). Using our benchmark dataset, we found that BdMitoCarta is more sensitive (correctly identifying 83% of known “Gold+” mitochondrial proteins) compared to *P. falciparum* (45% sensitivity) and *T. gondii* (72% sensitivity) but has a higher false discovery rate (13% versus 5% and 8%, respectively) (Figure S4A, Data S4). There is high overlap between the inventories (Figure S4B, S4C), with BdMitoCarta containing more unique proteins, consistent with its increased sensitivity. These unique proteins are ~10-fold lower abundance than the proteins annotated as mitochondrial in all three species (Figure S4D), indicating the power of our enrichment protocol coupled with DIA-MS to detect low-abundance proteins.

The high sequence divergence seen in parasite genomes poses a challenge for homology detection from *B. divergens* to other eukaryotic clades beyond Apicomplexa. Leveraging protein sequence, sequence profiles, and structural homology, we applied our orthogroup approach to species spanning 203 eukaryotic and 34 prokaryotic clades. We found that 52% of BdMitoCarta orthogroups showed homology to prokaryotes, 20% showed homology to eukaryotes, and 27% lacked detectable homologs outside SAR (Figure 3C, Data S5). Our evolutionary modeling suggests that the majority of these apparent SAR-specific orthogroups (108/132) contain proteins that are too short or diverged to reliably detect homology outside of SAR (i.e., underpowered by abSENSE-HMM [Chen co-submitted]), which makes function prediction by sequence challenging. Indeed, most of these 132 apparent SAR-specific orthogroups lack any PFAM domain or functional annotations (Figure 3D) and will require further analyses to elucidate function. The remaining 36% of the apparent SAR-specific orthogroups could be assigned putative functions based on the presence of PFAM domains or homology to *T. gondii* or *Plasmodium* proteins with known functions (Figure 3D). Many of these proteins are predicted to be OXPHOS components. This reflects the known biology of apicomplexan OXPHOS complexes, which are known to be expanded, containing many subunits not found in the corresponding complexes of humans and yeast.^5,22,23,46–48^

### *B. divergens* mitoproteome is small and highly reduced

*B. divergens* has the smallest mitoproteome of any bona fide mitochondrion (as opposed to a mitochondrial remnant organelle) characterized in the MitoCarta Tree of Life project (Figure 4A). Based on MitoTOL, we identified 803 mitochondrial orthogroups that are shared in at least two evolutionarily distant eukaryotic clades, which we use here as a simple proxy for being “ancestral” (Data S5A). Since 526/803 (66%) of these likely ancestral protein families are absent from BdMitoCarta (Figure 4A), this suggests the *B. divergens* mitochondrion is highly reduced relative to the ancestral mitochondrion. The same conclusion is obtained from a broader and systematic evolutionary reconstruction of the mitoproteome of the last eukaryotic common ancestor (LECA) and modeling of subsequent gains and losses (Chen et al, cosubmitted). We detect ~200 mitochondrial protein families that are unique to *B. divergens* relative to other experimentally defined MitoTOL inventories (Figure 4A), and these likely represent a mixture of true cladespecific innovations, as well as ancestral proteins too diverged for homology detection. Many of these protein families are unannotated, presenting exciting avenues for future studies (Figure 4B).

To spotlight pathways that have been retained or not detected in *B. divergens*, we relied on human MitoPathway annotations (Figure 4C, Data S5D).^49^ This analysis, again limited to the 803 protein families shared in at least two MitoTOL inventories and thus likely ancestral, shows that *B. divergens* has potentially lost or relocated many pathways spanning metabolism, the mitochondrial central dogma, OXPHOS, and mitochondrial dynamics (Figure 4D). This analysis spotlights the loss of macromolecular complex I of the ETC, which is known to be compensated by NDH2 in apicomplexan organisms.^5,6^ It is interesting to note that although CI has been lost in Apicomplexa, some CI assembly factors remain (Figure 4D). Based on manual annotation, these proteins are either dual-functioning proteins (homologs of human AIFM3, FOXRED1, and NUBP1), or proteins that are repurposed for assembly of other OXPHOS complexes (Bdiv_003830c, which is homologous to an ATP synthase-associated protein in *P. falciparum* and *T. gondii*).^22,23^

Other likely losses occur in the mitochondrial central dogma pathways. There are no detectable homologs in *B. divergens* for any human mtDNA repair/stability enzyme. *Babesia* spp. have a linear, highly reduced mtDNA genome that would likely require different maintenance and replication machinery compared to the circular mtDNA of humans.^7,8,33^ Additionally, homologs for all but one tRNA synthetase (phenylalanine-tRNA synthetase) are missing from BdMitoCarta, likely because functionally active tRNAs are imported into mitochondria from the cytosol in apicomplexans.^50^ Homologs for many small molecule and ion transporters, including sideroflexins and the calcium uniporter, are not detected in BdMitoCarta. *B. divergens* also lacks detectable homologs for the carnitine shuttle, which transports fatty acids into the mitochondrial matrix for catabolism. Other pathways linked to fatty acid catabolism (propanoate and vitamin B12 metabolism) and fatty acid oxidation also appear absent from BdMitoCarta. For these and other pathways that are apparently absent in BdMitoCarta, future work is needed to distinguish between true losses and protein divergence beyond the point of homology detection.

The pathways that are most conserved between *B. divergens* and humans are Fe-S cluster biosynthesis, protein import, and chaperone-mediated folding. In fact, these appear to be the most conserved mitochondrial pathways across the eukaryotic tree of life, being found even in mitochondrial remnant organelles (Chen, co-submitted).

### Evidence for metabolic remodeling in *B. divergens*

Like other apicomplexans, *B. divergens* metabolite transport and the TCA cycle diverge from the canonical animal pathway (Figure 5A). Pyruvate dehydrogenase complex (PDH) is absent and thought to be replaced by the branched-chain alpha-keto acid dehydrogenase (BCKDH)^51^ (4/4 subunits in BdMitoCarta). “Span I” TCA cycle enzymes are present, including citrate synthase, aconitase, isocitrate dehydrogenase (IDH), oxoglutarate dehydrogenase (OGDH), succinyl-CoA synthetase, and succinate dehydrogenase. Subsequent “span II” TCA cycle enzymes, however, differ from the human TCA cycle and include a malate-quinone oxidoreductase (MQO) to replace malate dehydrogenase and class I fumarate hydratase (FH) containing an Fe-S cluster.

The small molecule carriers and channels present in the BdMitoCarta inventory can further inform metabolic functions of the mitochondrion (Figure 5A). BdMitoCarta contains ion transporters (VDACs and LETM1 homologs), the pyruvate transporter (MPC homologs), and adenine nucleotide translocator (ANT) proteins. Homology allowed us to predict cargo for many SLC25A carrier proteins (for NAD^+^, iron, phosphate, oxoglutarate/malate, glutathione, and S-adenosyl-L-methionine (SAM)/S-adenosyl-L-homocysteine (SAH)). Similarly, homology let us predict one ABCB family transporter related to Fe-S cluster transport, but two additional ABCB transporters remain uncharacterized.

### *B. divergens* has two IDH homologs

Unexpectedly, we found the *B. divergens* mitochondrion to possess two NADP^+^-dependent isocitrate dehydrogenase (IDH) homologs: Bdiv_034270c (which we term BdIDH) and Bdiv_038090 (which we term BdIDHL for “IDH-like”). Both homologs showed clear enrichment in our mitochondrial fractions for both replicates (Data S3) and have a mitochondrial localization prediction (DeepLoc2.1). BdIDH appeared to be four-times higher abundance than BdIDHL in whole cells (iBAQ, Data S2). Microscopy analysis showed dual localization of BdIDH to the mitochondrion and another compartment, and mitochondrial-like localization for BdIDHL, with signal frequently appearing directly adjacent to that of the mitochondrial matrix marker HSP60 (Figure 2C, S3).

We traced the evolutionary distribution of these IDH homologs. *P. falciparum* has one IDH homolog (localized to the mitochondrion) and *T. gondii* has two IDH homologs (one mitochondrial and one apicoplast-localized).^52^ Phylogenetic analysis of 1,125 IDH domain-containing proteins across >200 eukaryotes and >20 prokaryotes demonstrated that NADP^+^-dependent IDH homologs clustered into a prokaryotic clade and a eukaryotic clade (Figure 5B). We note that apicomplexans contain only NADP^+^-dependent (not NAD^+^-dependent) IDH enzymes^13^. While BdIDH clustered in the expected location in the gene tree, we were surprised to observe a clade comprised of Bdiv_038090, three additional apicomplexan proteins (from *Gregarina niphandrodes*, *B. microti*, and *Babesia sp. Xinjiang)*, and a protein from the metamonad *Barthelona* located near the root of the eukaryotic clade and distinct from all other NADP^+^-dependent homologs. *B. duncani* was not included in the phylogenetic analysis, but synteny analysis revealed an additional Bdiv_038090 homolog in this species (BdWA1_001933) (Figure S5A). *B. bovis* and *B. bigemina* reference proteomes do not possess Bdiv_038090 homologs.

Given the unique phylogenetic relationship of the two *B. divergens* IDH homologs, we generated protein structural models with AlphaFold3^53^ and compared the predicted cofactor and substrate binding sites to the crystal structure of IDH2, the mitochondrial NADP^+^-dependent enzyme in humans. As expected from the phylogenetic analysis, human IDH2 and BdIDH share many conserved residues for binding of the NADP^+^ cofactor, including an arginine and histidine to coordinate the adenosine 2’-phosphate group, as well as residues for binding the required magnesium cofactor and substrate, isocitrate (Figure 5C, Figure S5B). In contrast, BdIDHL has the residues for binding magnesium but lacks two of the three arginine residues involved in binding isocitrate and lacks the arginine and histidine needed for binding the NADP^+^ phosphate (Figure 5C, Figure S5B). Further, it contains a tryptophan residue within the putative NADP^+^ binding pocket that would clash with the adenine ring of NADP^+^. This strongly suggests that BdIDHL would not bind either NADP^+^ or NAD^+^.

We recombinantly expressed and purified BdIDH and BdIDHL in bacteria. Both proteins eluted from a gel filtration column at volumes corresponding to the molecular weight of homodimers (Figure 5D). We observed NADP^+^-dependent isocitrate dehydrogenase activity for BdIDH (Figure 5E) with kinetics consistent with what has been reported for other NADP^+^-dependent isocitrate dehydrogenases (Figure 5F). In contrast, we could not detect any isocitrate dehydrogenase activity using either NAD^+^ or NADP^+^ as cofactors for BdIDHL (Figure 5E). Future work is needed to determine if BdIDHL catalyzes an oxidation reaction using an alternative redox cofactor, catalyzes an entirely different reaction type, or possibly regulates BdIDH activity similarly to what is observed in many NAD^+^-dependent IDHs.^54^

### Complexome analysis of the BdMitoCarta

Blue Native PAGE (BN-PAGE) is one of the classical methods for characterizing large macromolecular complexes in mitochondria. Protein compositions of *P. falciparum* and *T. gondii* OXPHOS complexes have been characterized using BN-PAGE,^22,23^ revealing unusual characteristics of the apicomplexan OXPHOS system, including expansion of ETC complexes (CII-V). Although we know atovaquone inhibits CIII and is used as a first-line therapy for babesiosis,^31,32^ the composition of OXPHOS complexes in *Babesia* spp. has yet to be experimentally defined.

Beginning from the mitochondria enrichment protocol developed here, we carried out BN-PAGE complexome analysis. To assess the intactness of our mitochondrial complexes, we performed an in-gel activity assay for cytochrome c oxidase (CIV) using mitochondria isolated from *B. divergens* and bovine hearts. This assay revealed CIV activity in BN-PAGE-separated mitochondrial extracts from both organisms and highlighted a high molecular weight CIV in *B. divergens* (Figure 6A) consistent with reports in other apicomplexans.^22,55^

We then performed BN-PAGE proteomic analysis on *B. divergens* mitochondria solubilized using either DDM (0.5%) or digitonin (0.75%). Use of both detergents can provide valuable information, as they differ slightly in their ability to solubilize and resolve complexes and supercomplexes. We cut each gel into 60 slices for analysis by mass spectrometry. When investigating the presence and composition of protein complexes, we limited our analysis to BdMitoCarta proteins (507 and 513 proteins detected in the DDM- and digitonin-solubilized samples, respectively) (Figure 6B, Data S6). We note that apparent molecular weights on BN-PAGE are notoriously imprecise, both because of the mass of the detergent-protein micelles and the shape of the protein complex. We estimated approximate molecular weights based on previously-characterized bovine OXPHOS complex standards in a parallel gel lane, with caveats that these estimates are outside of the linear range >2,000 MDa (slice numbers above 44) (Figure 6B, Data S6D).

Our complexome analysis allowed us to simultaneously consider co-migration as well as subunit iBAQ abundance when investigating protein complex composition. True complex members with stoichiometric relationships tend to have comparable abundances, whereas transiently bound proteins or those that co-migrate by chance are typically much less abundant. For instance, we observe that complex III (CIII) subunits migrate specifically at slice 35 following digitonin solubilization but are several orders of magnitude more abundant than subunits of CII and CIV which also peak in this slice (Figure 6C, Figure S6). The two detergents also provide specificity in this case, since when DDM is used for solubilization, CIII peaks in slice 34 (unlike CII and CIV) (Figure S6). iBAQ estimates suggest that CIII, OGDH, and BCKDH are amongst the most abundant complexes in the mitoproteome (Figure 6C).

Based on manual annotation via homology, we observe co-migration of 14 different complexes in the BN-PAGE analysis (Figure 5D). Specifically, a total of 186 proteins within these 14 different complexes showed co-migration, with 98% showing co-migration in both detergents. We observe highly specific migration patterns for all four OXPHOS complexes (CII, CIII, CIV, CV), the mitochondrial ribosome large (MRPL) and small (MRPS) complexes, abundant TCA cycle complexes (BCKDH, OGDH), and prohibitin (PHB). We observed striking similarity in the migration patterns of CII and CIV, with two different peaks observed in each detergent (slice 37 and 35 in DDM, 45 and 35 in digitonin, Figure S6). This suggests the possibility of either a surprising CII:CIV supercomplex, or perhaps similar multimer assemblies that happen to co-migrate by chance. Further studies are required to distinguish these possibilities.

Although the structure of the mitochondrial ribosome complexes have been elucidated by CryoEM in *T. gondii*,^56,57^ these complexes were not observed in previous BN-PAGE analyses in *T. gondii*^23^ or *P.* falciparum.^22^ Here, each detergent captured and showed strong co-migration of MRPS (44/49 subunits mapped by homology) and MRPL (70/75 subunits mapped by homology) (Figure S7, Data S6). Mapped MRPL proteins all show a coherent pattern with a single peak at slice 51 in both detergents. In contrast, MRPS subunits show four different co-migration patterns that might represent assembly intermediates, with most peaking at slice 46 and 45 and a subset also showing migration with MRPL at slice 51 (Figure S7).

To identify unannotated components or assembly factors of OXPHOS or the mitoribosome, we searched for proteins that co-migrated with each annotated complex (Figure 6E). We first selected co-migrating subunits of each complex as “baits”, created a mean bait profile, and used Pearson correlation across all slices to identify co-migrating candidates for each detergent, separately. We then looked for candidates with high scores in both detergents. This procedure identified two co-migration candidates for CV, seven for MRPL (one paralog to a known subunit, five with mitoribosome assembly/binding domains, and one additional candidate) and four for different bait sets from MRPS (Figure 6E, Figure S6, Figure S7, Data S6). Interestingly, the two co-migration candidates for CV lack homologs in *T. gondii* and *P. falciparum*, suggesting *Babesia*-specific biology. Co-migrating proteins and baits showed roughly similar abundance for CV and MRPS, suggesting core complex membership. However, co-migration hits showed lower abundance for MRPL, suggesting the possibility of transiently bound assembly factors (Figure S8). As expected, most of these co-migrating proteins lack homologs in apicomplexan species that also lack mtDNA, OXPHOS, and mitoribosomes (Figure S8). Future studies will be required to determine if these new co-migrating proteins, highlighted in green in Figure 6E, represent new complex subunits/assembly factors, conserved subunits that have diverged too rapidly for homology detection, or proteins co-migrating by chance.

## DISCUSSION

Study of apicomplexan mitochondria is significant from the perspectives of public health relevance, as well as eukaryote evolution; yet these systems are challenging for experimental investigation. During evolution, these mitochondria have remodeled to closely integrate with the apicoplast and host pathways and are essential for survival, making them excellent drug targets. Yet these same features complicate systematic investigation of their mitochondrial machinery with high experimental accuracy. Here we have developed an experimental workflow for enriching the single mitochondrion of *B. divergens* and used protein correlation profiling to define a high confidence mitochondrial protein inventory. To the best of our knowledge, the BdMitoCarta is the most comprehensive mitochondrial proteome for any apicomplexan. We anticipate that the workflow we have devised, and the proteome and complexome resources, will be broadly useful to the Apicomplexa research community.

Isolating enriched fractions of intact mitochondria proved extremely challenging and required optimizing multiple steps in our workflow. Since *Babesia* spp. replicate intracellularly, it was necessary to first remove the parasite from the host. Although saponin treatment is commonly used for lysing *Plasmodium*- or *Babesia*-infected erythrocytes, it resulted in lower downstream organellar recovery. We found filtration to be the best method for achieving erythrocyte lysis and parasite extrusion from the host cell. The addition of Benzonase nuclease following gentle parasite lysis was important, likely because DNA released (from nuclear rupture) aggregates with proteins and organelles. Mitochondria are fragile, so we minimized the pelleting and resuspension steps, e.g., an OptiPrep cushion allowed us to concentrate the sample without having to pellet. To avoid mitochondrial rupture and degradation, the protocol was completed as quickly as possible using fresh (not frozen) cultures. Each of these individual steps contributed to the success of our enrichment protocol. DIA-MS allowed us to profile each of the fractions at high depth of coverage without an excess of “missing values.” The resulting dataset analyzed by protein correlation profiling yielded a high-confidence BdMitoCarta inventory. Of note, the experimental workflow and PCP analysis recovered all three mtDNA-encoded proteins, which are notoriously difficult to detect experimentally, and provides experimental evidence that these three genes continue to be translated.

Our *B. divergens* total proteome and MitoCarta inventories highlight key metabolic pathways that *B. divergens* has selectively lost among apicomplexan parasites. Namely, *B. divergens* does not have enzymes for heme, fatty acid, or lipoate biosynthesis. In vertebrates, *T. gondii* replicates within nucleated cells and *Plasmodium* spp. replicate in hepatocytes and erythrocytes. Perhaps related to loss of the heme pathway, *Babesia* spp. exhibit a more narrow tropism within their mammalian hosts compared to their apicomplexan cousins, only replicating within heme-rich erythrocytes.

*B. divergens*, like other apicomplexans, has a TCA cycle that is very different from the canonical pathway in animals and fungi. Apicomplexan mitochondria lack a pyruvate dehydrogenase complex and the canonical fumarase and malate dehydrogenase are replaced by a class I fumarase (containing an Fe-S cluster) and a malate:quinone oxidoreductase (that directly feeds reducing equivalents into the ETC), respectively. In contrast to other characterized apicomplexan mitoproteomes, BdMitoCarta contains two IDH homologs, which we now designate BdIDH and BdIDHL. *In vitro* enzyme assays confirmed that BdIDH is a *bona fide* isocitrate dehydrogenase. BdIDHL (Bdiv_038090) also purified as a homodimer but had no IDH activity and was deemed unlikely to bind either NAD^+^ or NADP^+^ due to a steric clash within the predicted cofactor binding pocket. BdIDHL is conserved in other *Babesia* species, including *B. microti*, *Babesia sp. Xinjiang, and B. duncani.*, indicating it was an early innovation in *Babesia* spp. A future challenge lies in defining the function(s) of BdIDHL and understanding the selective advantage its presence may confer.

In addition to generating the mitoproteome of this organism, we have also generated a companion mitochondrial complexome using two different detergent conditions. In each of our two complexome datasets, we detected nearly all the BdMitoCarta proteins, and we observed co-migration of 186 BdMitoCarta proteins within 14 different complexes. We observe co-migration of all ETC CII and CIV subunits raising the possibility of a CII:CIV supercomplex, unless these complexes each have multimer assemblies that happen to co-migrate by chance. We also identify a small number of proteins showing clear co-migration with complex V (two proteins), the small mitoribosome (four proteins) and the large mitoribosome (one novel protein). The complexome co-migration was particularly useful to map mitoribosome subunits, which are notoriously difficult to identify via homology as they include many short and rapidly evolving proteins (e.g. approximately half of the human and yeast protein subunits lack similarity by BLASTP). Our complexome co-migration patterns helped resolve ambiguous homologs from the CryoEM-resolved subunits from *T. gondii*.^57^

We anticipate that the approaches presented here, with modification, can be applied more broadly to apicomplexans to systematically define the shared and unique machinery in this clade that is highly divergent from canonical systems. Such analysis will reveal conserved mechanisms for biochemical coupling with the apicoplast, while also defining lineage specific adaptations that support host tropism and survival in different life cycle stages. Further investigations will provide deep insights into principles of mitochondrial metabolic adaptation while also pointing the way to more effective medicines for diseases caused by *Babesia* spp. and other Apicomplexa pathogens.

### Limitations of the study

We acknowledge several limitations of the current study: 1) Since we are unable to synchronize *B. divergens*, the mitochondrial proteome we have obtained represents an average of a mixed population of asexual substages. Future research endeavors will focus on understanding how the mitoproteome shifts throughout the parasite lifecycle, both in terms of composition and protein abundances. 2) We benchmarked the sensitivity and specificity of our BdMitoCarta using proteins from *P. falciparum* with strong experimental evidence of mitochondrial localization. This approach is confounded if there are species-specific differences in the mitochondrial proteome. Indeed, our microscopy validation has confirmed that four of our benchmark proteins are not mitochondrial in *B. divergens.* 3) The genome annotations of *Babesia* species are regularly updated, which could impact our inventory. However, when updated genome annotations for *B. divergens* become available, we can generate new protein sequence databases to search our existing MS data against and update the BdMitoCarta inventory accordingly. Importantly, this will not require regenerating any samples for MS analysis. 4) Our PCP approach is limited with respect to dual localized proteins. 5) Remote homology detection continues to be challenging, especially for parasitic proteins, so proteins that are “absent” in *B. divergens* may be present but diverged beyond the point of recognition at the sequence level.

## RESOURCE AVAILABILITY

### Lead contact

Requests for resources and further information should be directed to and will be fulfilled by the lead contact, Manoj T. Duraisingh, (mduraisi@hsph.harvard.edu).

### Materials availability

All *Babesia divergens* strains and plasmids developed for this study are available from the lead contact (Manoj T. Duraisingh, mduraisi@hsph.harvard.edu), upon request.

## STAR★METHODS

### EXPERIMENTAL MODEL AND STUDY PARTICIPANT DETAILS

#### Cell culture.

*Babesia divergens* strain Rouen 1987 was maintained in purified O+ human male erythrocytes (RBCs, Research Blood Components) at 2% hematocrit in RPMI-1640 medium supplemented with 25 mM HEPES, 11.50 mg/L hypoxanthine, 2.42 mM sodium bicarbonate, and 4.31 mg/mL AlbuMAX II (Invitrogen). Every second day, the cells were either split, or a full media change was performed. Transfected cultures were supplemented with blasticidin (20 μg/mL) to maintain construct expression. Cultures were incubated at 37 °C within a hypoxic chamber (1% O_2_, 5% CO_2_).

### METHOD DETAILS

#### Molecular cloning of plasmids.

DNA constructs for MitoIP (3xHA-eGFP-TOM22 and 3xHA-eGFP) and MitoCarta inventory validation were generated by Twist Bioscience and cloned into ectopic circular DNA plasmids using NEBuilder HiFi DNA Assembly Master Mix (New England Biosciences Cat. #E2621). Plasmid pBE13 (Brendan Elsworth, HSPH) was used for the MitoIP constructs and plasmid 0mod-prime_pBE13 (generated by GenScript) was used for MitoCarta inventory validation constructs. Plasmid 0mod-prime_pBE13 was designed for this study to allow expression of *B. divergens* genes from their native promoters. The assembled plasmids were transformed into XL10-Gold ultracompetent *E. coli* (Aligent cat. #200315) to scale for parasite transfections. Plasmid preparations were sequenced via whole plasmid sequencing (Plasmidsaurus).

#### Plasmid transfections.

*B. divergens* parasites were cultured to 10%-20% parasitemia using previously described culture conditions. On the day of transfection, parasite cultures were pelleted by centrifugation (500 *g*, 5 min). The resulting pellet of infected erythrocyte culture (iRBCs) was resuspended to 10% hematocrit with complete RPMI-1640 growth media. The cell resuspension was separated into 500 μL aliquots, which were kept at 37 °C until ready for use. 10 μg of sterile plasmid material (1 μg/mL in nuclease-free sterile H_2_O) was combined with 18 μL of Supplement and 82 μL of P3 from a P3 Primary Cell 4D-Nucleofection Kit (Lonza Cat. #V4XP-3024). Aliquoted iRBCs were pelleted by centrifugation (500 *g*, 5 min), resuspended in the plasmid mixture, and added to an Amaxa Nucleocuvette Vessel for transfection in an Amaxa 4D-Nucleofector X Unit (Lonza Cat. #AAF-1003X). The FP158 program was selected for transfection of iRBCs. After transfection, the cuvette contents were added to 1 mL of growth medium and centrifuged (500 *g*, 1 min) to remove debris. The cell pellet was then resuspended in 1 mL of complete RPMI-1640 growth media containing 300 μL of uninfected erythrocytes. The cell resuspension was added to a petri dish containing 9 mL of complete RPMI-1640 growth media and incubated at 37 °C in a hypoxic chamber. One day after transfection, the spent media was replaced with fresh RPMI-1640 growth media containing 20 μg/mL blasticidin to begin selection. The cell culture media was replaced with fresh RPMI-1640 growth media containing 20 μg/mL blasticidin, daily for five days and then every other day until parasites were detectable by blood smear. Transfected parasite lines were cloned by limited dilution and plasmid carriage confirmed by PCR on genomic DNA extracts.

#### Sample preparation for immunofluorescence assay.

Parasite cultures for immunofluorescence assay (IFA) analysis were allowed to settle onto poly-D-lysine-coated coverslips (15 min, 37 °C). After settling, the cells were gently rinsed three times with 1x PBS and fixed with 4% paraformaldehyde (PFA) in 1x PBS (15 min, 37 °C). The cells were washed with 1x PBS (3x 5 min, rt), permeabilized with 0.1% Triton X-100 in 1x PBS (10 min, rt), and blocked with 3% bovine serum albumin (BSA) in 1x PBS (1 hr, rt). Primary antibody incubation was performed overnight (16-18 hr, 4 °C,) with rat anti-HA antibody (1:250 in block; Roche Cat. #11867423001) and rabbit anti-HSP60 antibody (1:1,000 in block; Novus Biologicals Cat. #NBP2-12734). The cells were washed with 1x PBS (3x 5 min, rt) and incubated (1 hr, rt, protected from light) with secondary antibody solution containing Alexa Fluor 488 goat anti-rat antibody (1:1,000 in block; Invitrogen Cat. #A-11001), Alexa Fluor 555 goat anti-rabbit (1:1,000 in block; Invitrogen Cat. #A-21428) and Hoechst (1:1,000 in block; Thermo Cat. #62249). The cells were washed with 1x PBS (3x 5 min, rt, protected from light) and the coverslips were mounted onto slides using VectaShield Vibrance Mounting Media (Vector Laboratories Cat. #H-1700).

#### Sample preparation for ultrastructure expansion microscopy.

Ultrastructure expansion microscopy (U-ExM) was performed on the parasites as previously described.^64^ Briefly, parasites were settled onto poly-D-lysine-coated coverslips in media containing 300 nM MitoTracker Orange CMTMRos (Thermo Cat. #M7510; 30 min, 37 °C, protected from light). All further steps were performed in the dark to prevent MitoTracker signal loss. The cells were fixed with 4% PFA (15 min, 37 °C), washed with 1x PBS (3x 5 min, rt), and incubated with 1.4% formaldehyde, 2% acrylamide solution in 1x PBS overnight (16-18 hr, 4 °C). After the overnight incubation, the treated coverslips were gelated by adding 35 μL of monomer solution (19% sodium acrylate, 10% acrylamide, 0.1% bis-acrylamide in 1x PBS) mixed with ammonium persulfate and TEMED. The gels were allowed to solidify (1 hr, 37 °C) prior to incubation with denaturation buffer (100 mM SDS, 200 mM NaCl, 50 mM Tris-HCl, pH 9.0) for 15 min on a rocker followed by 90 min at 95 °C. The gels were washed and allowed to expand overnight (16-18 hr, 4 °C) in MilliQ water. Following the overnight incubation, the gels were shrunk in 1x PBS and blocked with 3% BSA in 1x PBS (30 min, rt). Primary antibody incubation was performed overnight (16-18 hr, 4 °C) with anti-HA antibody (1:200 in block; ABCD Antibodies Cat. #ABCD_AF291, guinea pig) and rabbit anti-HSP60 antibody (1:500 in block; Novus Biologicals Cat. #NBP2-12734). The gels were washed with 0.5% Triton X-100 in 1x PBS (3x 5 min, rt) and incubated (2.5 hr, rt) with secondary antibody solution containing Alexa Fluor 488 goat anti-guinea pig antibody (1:1,000 in 1x PBS; Invitrogen Cat. #A-11073), Alexa Fluor 555 goat anti-rabbit (1:1,000 in 1x PBS; Invitrogen Cat. #A-21428), Alexa Fluor 405 NHS Ester (10 μg/mL in 1x PBS; Invitrogen Cat. #A30000) and SYTOX Deep Red (1:1,000 in 1x PBS; Invitrogen Cat. #S11381). The gels were expanded in MilliQ water and imaged using poly-D-lysine-coated glass-bottom imaging dishes (Cellvis).

#### Image acquisition.

Mounted coverslips and gels were imaged using the 1.4 NA 63x oil immersion objective on a Zeiss LSM-900 confocal microscope equipped with Airyscan 2 (Carl Ziess Microscopy). 16-bit (for IFA) and 8-bit (for U-ExM) super resolution z-stack images were acquired in Airyscan mode with a resolution of 40 nm/pixel and z-interval of 0.10 μm. Image acquisitions were captured unidirectionally, without averaging, with a master gain of 850. Raw images were processed using the “Airyscan Processing” protocol enabling the 3D processing mode on Zen Blue software (version 3.5) and analyzed using FIJI.^65^

#### Mitochondrial enrichment from *B. divergens*.

For mitochondrial isolation, asynchronous cultures of *B. divergens* stably expressing the 3xHA-eGFP-Tom22 plasmid were grown to 600 mL (replicate 1) or 800 mL (replicate 2) total cell culture volume. Expression of the mitochondrial-localized 3xHA-eGFP-Tom22 construct was maintained by inclusion of blasticidin (20 μg/mL) in the growth media. When the culture achieved high parasitemia (~20-30%), 80% of the media in each dish was replaced with fresh growth media and the hematocrit of each dish was increased from 2% to 4% by addition of new RBCs. The next day, after cells returned to ~20-30% parasitemia, the total cell culture volume was collected and centrifuged to pellet RBCs (500 *g*, 5 min). In total, 24 mL of packed RBC volume (~2.4 x 10^11^ cells) was used for replicate 1 and 32 mL of packed RBC volume (~3.2 x 10^11^ cells) was used for replicate 2.

To isolate *Babesia*, the RBC pellet was resuspended to 30% hematocrit in fresh growth media containing 1 mM ascorbate (to quench extracellular hemoglobin upon RBC lysis). The cell suspension was placed on ice and chilled for 30 min in a 4 ºC cold room. For the remainder of the protocol, samples were kept on ice, and all steps were performed in a 4 ºC cold room using chilled reagents and glassware. Filter stages were built using 25 mm Swinnex filter holders (Sigma Cat. #SX0002500) and 2.0 μm pore size, 25 mm diameter polycarbonate membrane filters (Sigma Cat. # TTTP02500). RBCs were mechanically lysed by passing the cell suspension twice through the 2.0 μm filter stage. For the first filter pass, a new filter stage was used for each 10 mL volume of RBC suspension. For the second filter pass, a new filter stage was used for each 40 mL volume of suspension. The filtrate was centrifuged (5,000 *g*, 3x 10 min., 4°C) to pellet parasites, un-lysed RBCs, and RBC membranes. The supernatant and top, loosely packed material made up of RBC membranes was removed from the pellet by pipetting. The remainder of the pellet, comprised of parasites, was resuspended in SHEP Buffer (0.28 M sucrose, 10 mM HEPES-KOH, pH 7.2, 0.2 mM EDTA, 1x cOmplete^™^ Protease Inhibitor Cocktail (Roche Cat. #65741000)). The pellet was washed three times by resuspending the cells in SHEP buffer, centrifuging to pellet (5,000 *g*, 5 min., 4 °C), removing the supernatant, and repeating the resuspension. The washed pellet was resuspended in 12 mL (replicate 1) or 16 mL (replicate 2) of SHEP Buffer, and 1% of the sample was aliquoted into separate microcentrifuge tubes for western blot and mass spectrometry analysis (“whole cell” sample).

The *B. divergens* resuspension was transferred to a cell disruption vessel (Parr Instrument Company Cat. #4639). 1,750 PSI of ultrapure nitrogen gas was slowly applied to the vessel, and the sample was incubated for 15 min to allow gas diffusion into the cells. The lysate was slowly eluted (~1-2 drops per second) into a conical tube containing 500 units (2 μL, replicate 1) or 667.5 units (2.67 μL, replicate 2) of Benzonase Nuclease (Sigma Cat. #E1014). The lysate was incubated with Benzonase for 20 min., inverting the tube every 5 min to mix. Un-lysed parasites and nuclei were cleared from the sample by one (replicate 1) or three (replicate 2) rounds of centrifugation (500 *g*, 10 min, 4 °C). After the centrifugation, the supernatant was transferred to a new tube, carefully avoiding the pellet of un-lysed parasites. 2% of the sample volume was collected in separate microcentrifuge tubes for western blot and mass spectrometry analysis (“crude mito” sample).

The total sample volume was brought to 60 mL (replicate 1) or 80 mL (replicate 2) with SHEP Buffer and incubated with 300 μL (replicate 1) or 400 μL (replicate 2) of μMACS^™^ anti-GFP microbeads (Miltenyi Cat. #130-091-125) for 1 hr, with gentle rocking agitation. Following incubation, the sample was passed through a 30 μm pre-separation filter (Miltenyi Cat. #130-041-407) into a buffer-equilibrated LS column (Miltenyi Cat. #130-042-401) attached to a QuadroMACS^™^ magnetic separator stand (Miltenyi Cat. #130-091-051). A separate pre-separation filter and LS column was used for each 10 mL aliquot of sample, and each sample was applied to its column in 4x 2.5 mL increments. The columns were washed with 10 mL of SHEP Buffer, applied in 4x 2.5 mL increments. Following the wash step, the columns were removed from the magnet and the immunocapture was eluted by applying 2.5 mL (replicate 1) or 2 mL (replicate 2) of SHEP Buffer to each column and gently eluting with a plunger. The elution was repeated using an additional volume of SHEP Buffer per column. All elution samples were pooled into the same conical tube, and the total sample volume was brought to 30 mL with SHEP Buffer. 5% of the sample was aliquoted into separate microcentrifuge tubes for western blot and mass spectrometry analysis (“MitoIP” sample). The Whole cell, crude mito, and MitoIP samples aliquoted for western blot and mass spectrometry analysis were brought to a total volume of 1.5 mL with SHEP Buffer and centrifuged to pellet (15,000 *g*, 10 min., 4 °C). The supernatant was carefully removed from each sample, and the pellets were snap-frozen in liquid nitrogen and stored at −80 °C for downstream analysis.

A 50% w/v iodixanol solution was prepared by diluting 12 mL of OptiPrep (Sigma, Cat. #D1556) with 2.4 mL of OptiPrep Diluent Solution (0.28 M sucrose, 60 mM HEPES-KOH, pH 7.2, 1.2 mM EDTA). 1 mL of the 50% w/v iodixanol solution was added to the bottom of three 14 x 89 mm Thinwall Polypropylene Tubes (Beckman/Fisher, Cat. #NC9336079). 9 mL of IP elution sample was gently overlayed onto each of the 50% w/v iodixanol “cushions”. The samples were ultracentrifuged using an SW 41 Ti (Beckman, Cat. #333790) swinging-bucket rotor (50,000 *g*, 30 min, 4 °C, max acceleration, deceleration 8). Following ultracentrifugation, 8 mL of the upper layer of each gradient was removed, avoiding the material at the interface between the two density gradient layers. Each tube was gently swirled to resuspend the cushioned material and combine the density layers to achieve a 25% w/v iodixanol solution. The resuspended cushioned material from each of the three ultracentrifuge tubes was pooled into a single tube (6 mL total volume). Solutions of 10%, 15%, and 20% w/v iodixanol were prepared by mixing the 50% iodixanol solution with SHEP Buffer, as follows:

**Table:** 

Fraction	50% Solution (mL)	SHEP (mL)
10%	1.2	4.8
15%	1.8	4.2
20%	2.4	3.6

A discontinuous density gradient was built over the resuspended cushioned material by slowly layering 1.5 mL of the 20%, 15%, 10%, and 0% (SHEP Buffer) w/v iodixanol solutions on top of the 25% iodixanol layer. The density gradient was ultracentrifuged using an SW 41 Ti (Beckman, Cat. #333790) swinging-bucket rotor (50,000 *g*, 60 min, 4 °C, max acceleration, deceleration 8). Following ultracentrifugation, bands were isolated from the gradient by puncturing the side of the tube using a 5 mL syringe and an 18 G needle, transferring band material to chilled 15 mL conical tubes. The total volume of each collection was brought to 3 mL with SHEP Buffer to dilute the density media. The collections were mixed by inversion and split into two microcentrifuge tubes for mass spectrometry and western blot analysis All samples were centrifuged to pellet the band material (15,000 *g*, 30 min, 4 °C). The supernatant was removed from each pellet, 1 mL of SHEP Buffer was added to wash, and the samples were centrifuged to re-pellet (15,000 *g*, 10 min., 4 °C). Following the wash, the supernatant was removed, and the sample pellets were snap-frozen in liquid nitrogen and stored at −80 °C for downstream analysis.

#### Protein quantitation and immunoblots.

Samples were lysed in 1x RIPA buffer (Sigma Cat. #20-188) containing 1 mM PMSF (Cell Signaling Cat. #8553) and 2x cOmplete^™^ Protease Inhibitor Cocktail (Roche Cat. # 65741000). The protein concentration of each sample was determined by BCA assay (Thermo Cat. #23225). Samples were diluted in lysis buffer to achieve equal protein concentrations prior to mixing each sample with LDS loading buffer (Thermo Cat. # NP0007) containing 10 mM dithiothreitol (DTT). Proteins were denatured by heating (95 °C, 30 min) and separated using SDS-PAGE (4-12% Tris-Glycine gel, 135 V, 60 min, rt). Proteins were transferred to a PVDF membrane using the Trans-Blot Turbo Transfer System (BioRad). PVDF membranes were blocked with EveryBlot Blocking Buffer (10 min, rt; BioRad Cat. #12010020) and incubated with primary antibody in blocking buffer (16-18 h, 4 °C). Following overnight incubation, the membranes were washed with TBST (3x 5 min, rt) and incubated with secondary antibody in blocking buffer (1 h, rt). The membranes were washed with TBST (3x 5 min, rt) prior to incubation with Clarity Western ECL Substrate (5 min, rt; BioRad Cat. #1705061). Chemiluminescent signal was captured by film.

The following antibodies were used in immunoblot analyses: anti-Cytochrome C (1:2,500 in blocking buffer; Abcam Cat. #ab13575), anti-HSP60 (1:2,500 in blocking buffer, Novus Biochem Cat. #NBP2-12734), anti-HA tag (1:2,500 in blocking buffer; Cell Signaling Cat. #3724S), anti-LDH (1:2,500 in EveryBlot Blocking Buffer; LSBIO Cat. #LS-C488831), anti-rabbit IgG HRP-linked Antibody (1:10,000 in blocking buffer, Cell Signaling Cat. #7074S), anti-mouse IgG HRP-linked Antibody (1:10,000 in blocking buffer, Sigma Cat. #GENA931).

#### Sample preparation for mass spectrometry analysis.

Samples were lysed in SDS lysis buffer (5% SDS, 50 mM TEAB pH 8.5, 2 mM MgCl_2_, 2μg/ml Aprotinin, 10 μg/mL Leupeptin, 1 mM PMSF) and incubated on ice. Lysates were incubated (15 min, rt) with 750 units of Benzonase nuclease (Thomas Scientific Cat. #E1014-25KU) to shear DNA prior to supernatant clearance by centrifugation (20,000 *g*, 10 min). Protein concentration was estimated via BCA assay. Reduction of disulfide bonds and alkylation of cysteines were carried out by addition of DTT to a final concentration of 5 mM followed by addition of 10 mM iodoacetamide (IAA) in the dark. Phosphoric acid was added to a final concentration of 1.2% v/v.

Acidified lysates were precipitated with 6x the sample volume of ice-cold S-Trap buffer (90% methanol, 100 mM TEAB), transferred to S-Trap cartridges and centrifuged. Proteins were washed 4 times with S-Trap buffer, centrifuged, and reconstituted in digestion buffer (50 mM TEAB with trypsin and LysC, each at 1:50 enzyme:substrate ratio). Digestion was carried out overnight followed by sequential elution steps of 50 mM TEAB, 0.1% FA, and 50% ACN/0.1% FA. Eluates were snap frozen and dried under vacuum.

Peptides were reconstituted in 1% FA and desalted with a 40 mg Sep-Pak tC18 96-well Plate (Waters, SKU:186002320). Briefly, C18 wells were conditioned stepwise with 100% ACN and 4x 0.1% TFA. Peptides were loaded onto the C18 wells and washed three times with 0.1% TFA, followed by 1% FA. Desalted peptides were then eluted sequentially with 50% ACN/ 0.1% FA. Eluates were snap frozen and centrifuged under vacuum until dry.

#### LC-MS/MS analysis.

Peptides from each sample were reconstituted in 3% ACN/0.1% FA for LC-MS/MS analysis and run on an Orbitrap Astral mass spectrometer (Thermo Scientific) coupled to a Vanquish Neo UHPLC system (Thermo Scientific). 500 ng of peptides from each sample were injected onto a PepSep C18 column (25 cm x 75 μm, 1.5 μm) (Bruker Daltonics). Peptides were separated on a linear 47.5 min gradient from 4.6% solvent B (99.9% ACN, 0.1% FA) to 24% solvent B before ramping up to 48% solvent B over 4 min. To wash the column, solvent B was increased to 72% for 7.5 minutes. MS1 spectra were measured with resolution of 240,000 at 200 m/z and a mass range from 300 to 1,800 m/z. The maximum injection time for MS1 and MS2 were set to 5 msec. The AGC targets for MS1 and MS2 were set to 2e6 and 5e5 respectively. MS2 scans covered a mass range of 380–980 m/z with an isolation window of 4 Th. Precursors were fragmented with higher-energy collisional dissociation (HCD), using a normalized collision energy (NCE) of 27%.

#### Mass spectrometry data processing.

Data were processed using Spectronaut software version 19. Data from 10 samples (5 samples from replicate 1 and 5 samples from replicate 2) were searched together against a combined database that included both *B. divergens* (PiroplasmaDB-58_Bdivergens1802A) and human (*Homo sapiens* RefSeq release 63, using longest transcript per Entrez gene) (Data S2). The search was carried out in direct DIA mode using quantification settings with a precursor Q-value cutoff of 0.01, a precursor PEP cutoff of 0.2, protein Q-value cutoff (experiment) of 0.01, a protein Q-value cutoff (run) of 0.05, and a protein PEP cutoff of 0.75. The MS/MS search included the fixed modification of Carbamidomethyl (C) and variable modifications, Oxidation (M), Acetyl (Protein N-term), Deamidation (N), and FormylMet (Protein N-term), with a maximum variable modification count of 5. Cleavage rules were set to “Trypsin/P” and 4 missed cleavages were allowed.

Data corresponding to human proteins were filtered out and then column sum normalization was applied across samples (Data S3): (normalized_intensity_g,s_ = scalingFactor * intensity_g,s_ /columnSum_s_, for protein group *g* and sample *s*, where columnSum_s_ is the sum of intensities of sample *s*, and scalingFactor = mean(columnSum_s_ for all *s*)). Next, protein groups were expanded such that normalized_intensity_*p,s*_= normalized_intensity_*g,s*_ for all proteins *p* in protein group *g*. Normalized intensities were log_10_-transformed and then MinProbAll imputation was performed (missing values were replaced with values drawn from a Gaussian with mean set to the 1^st^ percentile of values across all samples, and sigma set to the median of standard deviations of each protein across all samples). Z-scores were computed for each protein across all samples. PCP score was calculated as the Pearson correlation between the Z-score profile for each protein versus the mean of three mitochondrial markers (TOM40 Bdiv_015510c; HSP60 Bdiv_005420; ISCU Bdiv_029170). Genes with PCP score ≥ 0.96 from Replicate 1 were labeled “MitoViaPCP”.

#### Protein sequences and annotations.

Protein sequences and GO annotations were obtained from PiroplasmaDB-58_Bdivergens1802A, with the mitochondrial genome and annotation from *B. divergens* Rouen kindly provided pre-publication by Dr. Alejandro Sanchez-Flores.^66^ Protein orthologs were assigned using BLASTP^67^ best bidirectional hit (BBH) with E-value < 1e-3 to *P. falciparum* 3D7 PlasmoDB-58 and *T. gondii* ME49 (ToxoDB-62) and *Homo sapiens* (RefSeq release 63, using longest transcript per Entrez gene) as well as via orthogroups. *B. divergens* protein sequences were annotated with protein domains using HMMER version 3.1 (using the Pfam-A database version 35).^68^

#### Benchmarking dataset accuracy.

*B. divergens* genes were partitioned into three categories: “gold+” mitochondrial (n = 82 genes; based on BLASTP BBH to *P. falciparum* genes with strong evidence of mitochondrial localization from literature manual review), “possible_mito” (n = 843; the union of 750 genes predicted to be mitochondrial by DeepLoc2.1^69^ (score ≥ 0.4) and 443 genes with BLASTP E-value < 1e-3 to *P. falciparum* genes with PlasmoDB GO mitochondrial location), or “other“ (n = 3,207; all other genes, used as “gold-“ for benchmarking). Gold+ and gold− truth data were used to benchmark dataset accuracy as described in (^70^): true positives (TP), true negatives (TN), false positives (FP), false negatives (FN), sensitivity SN = TP / (TP + FN), specificity SP = TN / (TN + FP), corrected false discovery rate cFDR = (1-SP)/(1-SP + SN x *O_prior_*), and Precision = 1 – FDR. *O_prior_* = 0.08 prior probability of mitochondrial localization was assigned based on an estimated n = 450/5,389 mitochondrial proteins in *P. falciparum*.^19^ Based on this prior, we estimate “gold+” represents 25% of true mitochondrial proteins (0.18 = 82/(0.08*4133)) and “gold−“ represents 84% of true non-mitochondrial proteins (0.84 = 3207/(0.92*4133)) – thus it is imperative to correct for the sizes of our benchmark sets when estimating false discovery rates. Accuracy of each condition was assessed using log2fold-change(condition/whole cell) based on the first replicate in each condition, with missing values imputed as described above. The threshold of 0.96 for PCP analysis of replicate 1 was determined using maximum area under the precision-recall curve, and this same threshold was applied to replicate 2.

#### Orthogroup homology across the tree of life.

Homology across 203 eukaryotic species, 29 bacterial clades (grouped at superfamily-level), and 5 archaeal clades (grouped at kingdom-level) was determined based on orthologous groups (OGs) that were inferred by OrthoFinder (v2.5.5)^71^ and refined using a custom pipeline incorporating profile Hidden Markov Model and Alphafold structure alignments (Chen et al, co-submission; also see michaelzhuchen GitHub). Homology to additional apicomplexan species (not included in the 203 eukaryotic species) were obtained from the larger collection of 673 eukaryotic species used to create orthogroups. The species tree used for visualizations was inferred from a concatenated alignment of universal ribosomal proteins across eukaryotes, bacteria, and archaea using a manually refined NCBI taxonomy as a guide tree (Chen et al, co-submission; also see michaelzhuchen GitHub).

#### Comparison to hyperLOPIT studies.

Published hyperLOPIT tagm.map.allocation sub-cellular assignments from *T. gondii*^11^ (Tg) were mapped to *B. divergens* by BLASTP BBH (E-value < 1e-3). Clusters corresponding to the same organelle were combined for simplification (3 proteasome, 2 ER, 3 plasma membrane, 2 mitochondrion, 3 nucleus, 2 rhoptries). For Figure S4, orthogroups were used to assess homology in both Tg and *P. falciparum* (Pf), where Pf hyperLOPIT sub-cellular assignments^18^ were obtained using column “Final location (S1-S2)”. Benchmark analysis was performed on BdMitoCarta using benchmark data described above (82 gold+; 843 possible_mito; 3,207 gold−) and similarly for Pf (104 gold+; 870 possible_mito based on GO or DeepLoc2.1; 4,415 gold−) and Tg (89 gold+; 1,502 possible_mito; 6,731 gold−) using BLASTP BBH (E-value < 1e-3) to map annotations between species.

#### Essentiality analysis.

The 5,326 *P. knowlesi* proteins mapped to 3,891 unique OGs (Data S5), of which 98% (3,799/3,891) had a hybrid model essentiality score^45^. In the case of multiple *P. knowlesi* paralogs in the OG, the mean hybrid model essentiality score across the paralogs was used. Based on the thresholds in the original study, essentiality scores < 0.26 were deemed “essential”, yielding 1,476 OGs “essential” in *P. knowlesi* (1,476/3,797 = 39%, Figure 3B), of which 253 contained a BdMitoCarta member.

#### Determination of gene ancestry.

The putative ancestry of each protein was assigned based on the presence/absence of its OG across species. First, proteins with homologs in at least two prokaryotic clades were labeled as “Prokaryotic”. Second, proteins with homologs in at least two eukaryotic superfamilies (requiring homologs in at least two species within each superfamily) and in OGs containing >90% eukaryotic species were labeled as “Eukaryotic”. Third, proteins with no identified homologs beyond SAR were labeled as “SAR-specific”. Functional annotations for the SAR-specific orthogroups were assigned if any BdMitoCarta OG member had a PFAM domain or a gene description in PiroplasmaDB-58 (excluding “hypothetical protein” and “conserved hypothetical protein”).

#### Mitoproteome evolutionary comparison.

MitoTOL MitoCarta inventories were obtained from human,^49^ *Acanthamoeba castellanii* (Stefely et al, co-submitted), *Trypanosoma brucei* (Vacas et al, co-submitted), and *Arabidopsis thaliana* (Kim et al, co-submitted) and compared using OGs (Chen et al, co-submitted). We note that for simplicity, our analysis did not consider mitoproteomes from either *Leishmania tarentolae* (as it is too similar to *T. brucei*) and yeast (as it is possibly too similar to human as both are part of the Opisthokont clade). OGs were labeled as “Mito in >1 species” (contains MitoCarta members in at least 2 of these 5 species but may also have non-MitoCarta members in any species), or “Mito in 1 species” (contains MitoCarta members in exactly 1 of these 5 species but may also have non-MitoCarta members in any species) or “none”. We note that the 803 “Mito in >1 species” OG are likely to be ancestral, since ~85% of these are reconstructed to be present in LECA’s mitochondrial based on models using mitochondrial predictions from hundreds of eukaryote species (Chen et al, co-submitted). We downloaded human MitoPathways annotations^49^, where each human MitoCarta3.0 gene was manually assigned to one or more MitoPathways (147 hierarchical pathways) based on literature, and OGs were annotated with the union of MitoPathways from its human MitoCarta members.

#### Gene tree inference.

The IDH gene tree was inferred for a set of 1,125 proteins from any orthologous group that had an Iso_dh PFAM domain (HMMER;^68^ hmmsearch full sequence E-value < 0.001) across the set of 203 eukaryotes and additional apicomplexan species. A multiple sequence alignment was constructed using muscle5^72^ (v5.1, -super5) and trimmed by ClipKIT^73^ (v2.3.0, -m gappy). A maximum-likelihood phylogeny was inferred by IQ-TREE^74^ (v2.3.6, -m MFP -cmin 4 -cmax 12 -bb 1000 -alrt 1000 -seed 42 -wbtl -bnni).

#### Protein purification.

Codon optimized genes for Bdiv_034270c and Bdiv_038090 followed by a C-terminal GGS linker and 8X HIS-tag were synthesized and incorporated into separate pET-28a plasmids by TWIST Bioscience. BL21(DE3) Chemically competent *E. coli* (Invitrogen Cat. #C600003) were transformed with expression plasmid and grown in Luria-Bertani broth with 50 μg/mL kanamycin. Cells were grown at 37 °C until an optical density of 0.4 was reached, at which point the temperature was adjusted to 18 °C. At an optical density of 0.8, protein expression was induced with 0.5 mM IPTG and the cells were grown for an additional 16 hr, pelleted, and then frozen and stored at −80 °C.

Frozen cells were resuspended in resuspension buffer (RB; 50 mM HEPES pH 7.4, 200 mM NaCl, 4mM MgCl_2_, 10% glycerol) and sonicated after the addition of 1 mM PMSF, cOmplete^™^ Protease Inhibitor Cocktail, and lysonase. After centrifugation (40,000 *g*), the supernatant was run over Talon cobalt resin (Takara Cat. #635503) equilibrated with RB. The resin was washed with 10 column volumes of RB followed by 10 column volumes of RB with 20 mM imidazole and then protein was eluted with RB containing 300 mM imidazole. Protein was then concentrated and run over a HiLoad 16/600 Superdex 200 prep grade column (Cytiva Cat. #28989335). Purified constructs were analyzed by Coomassie staining of SDS-PAGE gels and identity confirmed by protein mass spectrometry at the Taplin Mass Spectrometry Facility at Harvard Medical School.

#### Steady-state enzyme kinetics.

Isocitrate dehydrogenase activity was measured with a Cary 100 spectrophotometer at a wavelength of 340 nm with a cuvette volume of 1.2 mL. Reactions were carried out in a buffer of 20 mM HEPES pH 7.4, 1 mM MgCl_2_, 1 mM NADP^+^ (or NAD^+^) and varying concentrations of potassium Ds-threo-isocitrate at 30°C. Reactions were initiated by addition of 5 nM enzyme. Normalized absorbance change over time was plotted with GraphPad Prism 11 (ver. 11.02.1). For steady state kinetic analysis an extinction coefficient of 6.22 mM^−1^cm^−1^ was used for calculating NADPH concentration. Initial rates were calculated with ICEKAT using the linear portion of the trace and k_cat_ and K_M_ were determined with GraphPad Prism 11 (ver. 11.02.1).

#### Sample preparation for Blue Native PAGE (BN-PAGE).

*B. divergens* mitochondria pellets were isolated by MitoIP as described above, then snap frozen in liquid nitrogen and stored at −80 °C. Two 40 μg samples were prepared: one sample solubilized in 0.75% w/v digitonin and the other solubilized in 0.5% w/v n-dodecyl-β-d-maltoside (DDM). For solubilization of each sample, MitoIP pellets were thawed at 4 °C on ice, and the following components added: NativePAGE 4x Sample Buffer (5 μL, Thermo Cat. #BN2008), 5% digitonin (3 μL, Thermo Cat. #BN2008) or 10% DDM (1 μL, Thermo Cat. #BN2008), Benzonase (0.8 μL, Sigma Cat. #E8263-5KU), 50 mM MgCl_2_ (0.8 μL), and dH_2_O to a final volume of 20 μL. The samples were incubated on ice (4 °C, 20 min) and then centrifuged (20,000 *g*, 10 min, 4 °C). The supernatant (19 μL) was transferred to a new tube on ice and a volume of NativePAGE 5% G-250 Sample Additive (Thermo Cat. #BN2008) equal to ¼ the detergent concentration was added. Two samples of bovine heart mitochondria (5.5 mg/mL, Abcam Cat. #110338) solubilized in either digitonin or DDM (according to the parameters above) were prepared in parallel.

Protein complex separation by BN-PAGE was performed in a cold room (4 °C), with the electrophoresis system (XCell SureLockMini-Cell) packed in ice. Protein complexes were separated on a NativePAGE Novex 3% to 12% Bis-Tris protein gel (Thermo Cat. #BN2011BX10). The inner gel chamber was filled with Dark Blue Cathode Buffer (1x NativePAGE Running Buffer (Thermo Cat. #BN2007), 1x NativePAGE Cathode Additive (Thermo Cat. #BN2007) in H_2_O) and the outer chamber was filled with Anode Buffer (1x NativePage Running Buffer in H_2_O). NativeMark Unstained Protein Standard (12 μL, Thermo Cat. #LC0725) and the samples (20 μL) were loaded into the gel wells, avoiding bubble formation. The gel was run for 30 min (150 V, 4 °C) prior to pausing the run and replacing the Dark Blue Cathode Buffer with Light Blue Cathode Buffer (1x NativePAGE Running Buffer, 0.1x NativePAGE Cathode Additive in H_2_O). The run was resumed for 150 min (250 V, 4 °C).

Following the run, the gel was removed from the cassette and fixed in Fixation Solution (40% (v/v) methanol, 10% (v/v) acetic acid, 10 mM ammonium acetate) (30 min, rt, with gentle rocking). The gel was de-stained in Destaining Solution (10% (v/v) acetic acid) (3x 20 min, rt, with gentle rocking) until the background became clear and the NativeMark Unstained Protein Standard could be visualized. The gel was washed in H_2_O (3x 10 min, rt, with gentle rocking) and digitally imaged with an Amersham Imager 680.

A custom template of 60 slices, upon which the gel could be superimposed, was prepared digitally and printed. The gel lanes of interest (DDM- and digitonin-solubilized *B. divergens* MitoIP samples) were cut vertically to separate from the rest of the gel. Then the pre-defined gel bands (60 slices) were cut horizontally across each lane individually. The individual gel bands were further cut into 5-6 small cubes, which were each transferred to a well of a 96-well plate filled with 150 μL of 50 mM ammonium bicarbonate. After each band collection, the razor and tweezers used for cutting and transferring samples were washed in methanol and water, to avoid cross-sample contamination. The band collection procedure was repeated for all 60 bands of each detergent condition. The fully loaded plates were stored at −20 °C prior to proteomic analysis.

#### BN-PAGE OXPHOS in-gel enzyme activity assays.

*B. divergens* MitoIP pellets and bovine heart mitochondria were solubilized in 0.5% w/v DDM and processed by BN-PAGE, as described in the previous section. Following the run, the gel was washed in ice-cold H_2_O and incubated with 10 mL Complex IV activity solution (0.05 mM cytochrome c, 2.3 mM diaminobenzidine, 50 mM sodium phosphate pH 7.2) (overnight, rt, gentle rocking), until brown bands appeared, then with 10% acetic acid to stop activity. To visualize the molecular weight ladder, the gel lane was stained with Coomassie Brilliant Blue R-250 stain solution (0.1% (w/v) Coomassie Brilliant Blue R-250, 25% (v/v) isopropanol, 10% (v/v) acetic acid) (30 min, rt, gentle rocking), then destained with 10% acetic acid until the ladder was visible (3 hr, rt, gentle rocking). The gel was digitally imaged with an Amersham Imager 680.

#### BN-PAGE gel slice processing and LC-MS/MS analysis.

Gel bands were destained by incubating with 100 μL of 50% acetonitrile (ACN) (15 min, rt, 400 rpm agitation). The solution was removed, and bands were washed with 100 μL of 50 mM ammonium bicarbonate (15 min, rt, 400 rpm agitation). The destaining and washing cycles were repeated three times until gel bands were clear. For protein reduction and alkylation, samples were treated with 50 μL of 10 mM dithiothreitol (DTT) at (45 min, 37 °C, 400 rpm agitation), followed by carbamidomethylation using 50 μL of 55 mM iodoacetamide (1 hr, rt, protected from light). Samples were washed sequentially with 50% ACN and ammonium bicarbonate, as described above. Gel bands were dehydrated with 100 μL of 100% ACN (10 min, rt, gentle shaking). After removing the ACN, gel pieces were rehydrated with 10 μL of trypsin (Promega, 5 ng/μL, 5 min, 37 °C). An additional 50 μL of ammonium bicarbonate was added to cover the gel pieces, and proteolytic digestion was carried out overnight (37 °C, 600 rpm agitation). The following day, supernatants were transferred to 0.5 mL micronic vials and peptides were extracted from gel pieces through sequential incubations with: 50 μL HPLC-grade water (400 rpm, 20 min, rt), 50 μL of 30% ACN/0.1% formic acid (FA) (400 rpm, 20 min, rt), and 50 μL of 70% ACN/0.1% FA (400 rpm, 20 min, rt). Supernatants were pooled, frozen, and lyophilized for subsequent analysis.

Samples were reconstituted in 45 μL of 0.1% FA and 20 μL were loaded on Evotip (Evosep Biosystems Cat. #EV2011) following standard procedure. Samples were run on an Evosep One (Evosep Biosystems) connected to a Q Exactive^™^ Plus Hybrid Quadrupole-Orbitrap^™^ Mass Spectrometer (Thermo). Peptides were separated on an Endurance Column (15 cm × 0.15 mm, 1.9 μm, Evosep Biosystems Cat. #EV1106) using the standard 30SPD method.

Samples were analyzed in data-dependent mode with an MS1 scan range of 300–1,800 m/z at 70,000 resolution. The automatic gain control (AGC) target was set to 3E6 ions and the maximum injection time was set to 50 ms. For each MS1 scan, the top 12 most abundant peaks were picked for fragmentation using a collision energy of 25 and isolation window of 1.7 m/z. MS2 spectra were acquired in centroid mode at a resolution of 17,500 with an AGC target of 5E4 and maximum injection time of 100 ms. Dynamic exclusion was set to 10 s. Ions were excluded if the charge was unassigned, equal to 1 or greater than 6.

#### BN-PAGE-MS data processing.

Spectrum Mill (Broad Institute) was used for mass spectrometry data processing. During raw file extraction, spectra within a precursor mass range of 600–6,000 Da and a minimum MS1 signal-to-noise ratio of 25 were retained. If MS1 spectra were within a retention time range of +/− 45 s, or within a precursor m/z tolerance of +/− 1.4 m/z, they were merged. MS/MS searching was performed against the *B. divergens* (PiroplasmaDB-58_Bdivergens1802A) proteome database. Digestion parameters were set to “trypsin allow P”, with an allowance of up to 4 missed cleavages. The MS/MS search included fixed modification of carbamidomethylation on cysteine and variable modifications (acetylation of the protein N-terminus, oxidation of methionine, conversion of glutamine to pyro-glutamic acid, deamidation of asparagine and conversion of carbamidomethylated cysteines to pyrocarbamidomethyl cysteines). Parent and fragment mass tolerances were set at 20 ppm. A maximum FDR threshold of 1% was used for peptide match validation.

#### BN–PAGE-MS data analysis.

We estimated the apparent molecular weight (MW) of each BN-PAGE gel slice using a linear model generated from a set of known MW markers from bovine heart mitochondria samples, which were processed and run in parallel with each *B. divergens* MitoIP lane. Protein groups detected in each slice were expanded such that all proteins in the protein group receiving the same top score (thus indistinguishable peptide evidence) were considered as detected. Relative abundance was calculated for each protein as iBAQ value / Max iBAQ value observed across any slice 1–60 (for digitonin) or slice 1-59 (for DDM, since top slice 60 contained a mixture of many complexes that apparently did not enter the gel). Heatmaps in Figure 6B are limited to BdMitoCarta proteins and were generated in Excel using the protein data in Data S6C. Complexes were manually annotated based on BLASTP BBH to literature in *P. falciparum* and *T. gondii*, and in cases where no BBH was available BLASTP Top Hit and OG candidates were reviewed. To identify co-migration candidates for each known protein complex, we (i) manually identified “bait” proteins that showed co-migration patterns in both DDM and digitonin, (ii) created a “bait” profile as the average of the relative intensity of these bait proteins in the 60 digitonin slices and separately in the 59 DDM slices, (iii) scored all other proteins using Pearson correlation to the “bait” profile to each experiment, (iv) manually reviewed high-scoring proteins (correlation > 0.9) to identify proteins showing comigration in both detergents. Figure 6C was generated in R using iBAQ values for the first protein per protein group (unlike the rest of Figure 6 which shows relative abundance per protein), after filtering out iBAQ values corresponding to relative abundance < 0.9. Figure S6,7 shows gene names based on homology to *T. gondii* and/or *P. falciparum*.

### QUANTIFICATION AND STATISTICAL ANALYSIS

Statistical analyses were performed in Excel and R. Statistical details can be found in the figure legends. Details of the benchmark analysis of published and newly generated mitochondrial proteomics datasets are provided in the Methods and include how benchmark datasets were created, corrected for size, and used to determine the sensitivity and precision of the MitoViaPCP list and previously published lists.

## Supplementary Material

1 Document S1. Figures S1–S8 and supplemental references.

2 Data S1. MitoCarta Tree of Life (MitoTOL) consortium membership roster and co-submitted MitoTOL manuscripts.

3 Data S2. DIA-MS intensity and iBAQ values for protein groups (A) and peptides (B) corresponding to the mitochondrial serial purification from *Babesia divergens*. Related to Figure 1.

4 Data S3. Mitochondrial Protein Correlation Profiling (PCP) results and annotations for all genes (A), the MitoViaPCP list (B), and BdMitoCarta (C). Related to Figures 1–5 and S2.

5 Data S4. Benchmark statistics corresponding to receiver-operator curves (ROC) and sensitivity and precision estimates for Figure 1H (A), Figure S2E (B), and Figure S4A (C). Related to Figures 1, S2, and S4.

6 Data S5. Orthologous group (OG) assignment for *Babesia divergens* and select eukaryotes (A), the subset associated with BdMitoCarta OGs (B), comparison with hyperLOPIT studies (C), and human MitoPathway annotations for OGs present or absent in *Babesia divergens* (D). Related to Figures 3, 4, and S4.

7 Data S6. Total intensity and iBAQ quantification for BN-PAGE complexome datasets in DDM (A) and Digitonin (B), plus an integrated dataset with relative intensities (C) and mapping of slice identifier to estimated molecular weight (D), and heatmaps for OXPHOS (E), MRPS (F), and MRPL (G) complexes. Related to Figures 6, S6, S7, and S8.

## Figures and Tables

**Figure 1.**
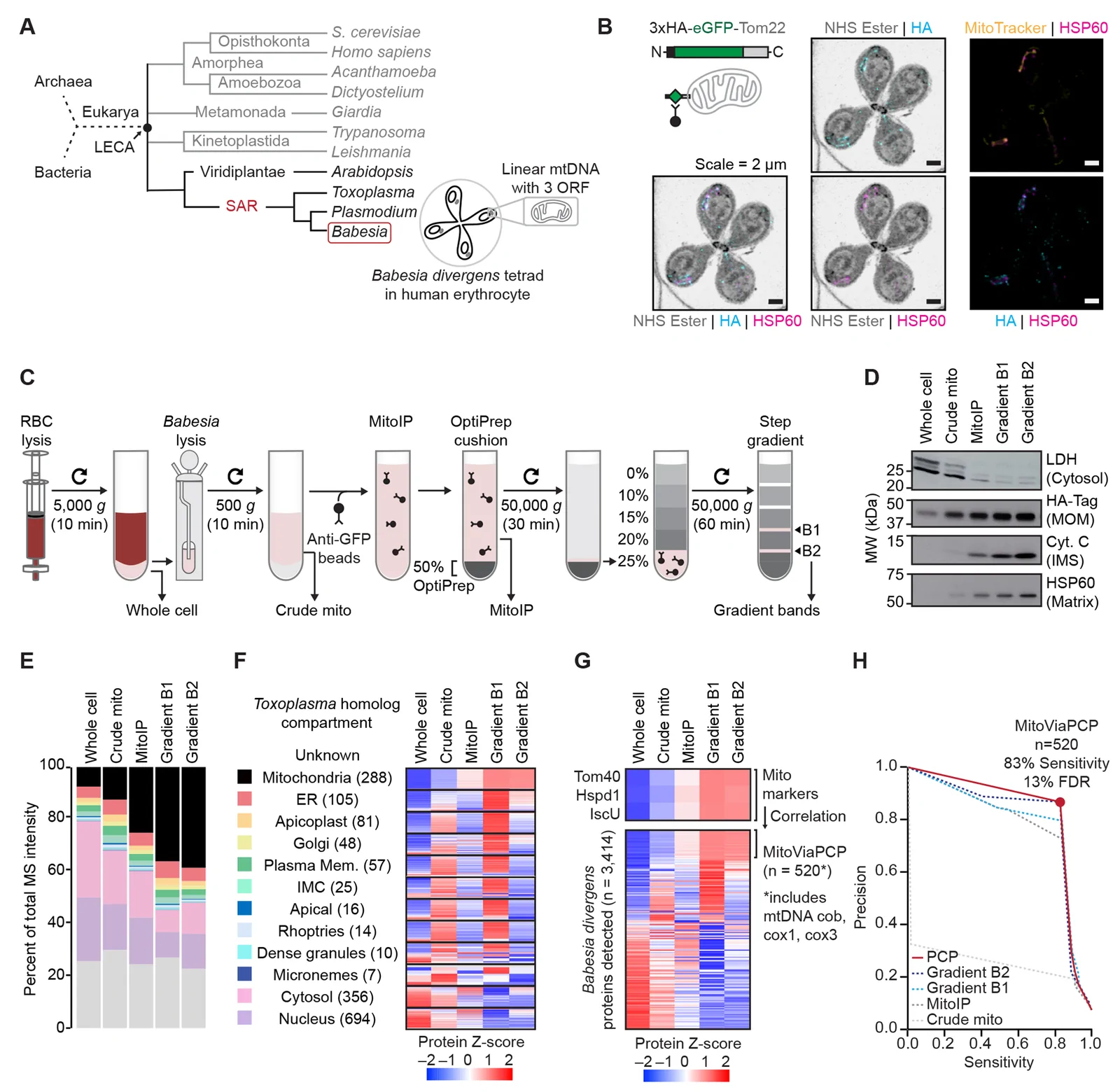
Mitochondrial protein inventory created via immunoprecipitation, density gradient separation, and protein correlation profiling (PCP). (A) Evolutionary position of *Babesia* spp. within phylum Apicomplexa of the Stramenopiles, Alveolates, and Rhizaria (SAR) supergroup, with cartoon depiction of organism. (B) Cartoon depicting the 3xHA-eGFP-BdTOM22 construct structure, and its validated localization to the mitochondrion via expansion microscopy analysis. All five images depict the same tetrad *B. divergens* merozoite (with mitochondrial markers MitoTracker and HSP60). Scale 2 μm. (C) Schematic overview of the mitochondrial enrichment protocol. Arrows indicate five fractions (Whole cell, Crude mito, MitoIP, and Gradient bands B1 and B2) collected for western blot and MS analysis. (D) Western blot analysis using antibodies against proteins localized to the cytosol and three mitochondrial compartments: mitochondrial outer membrane (MOM), intermembrane space (IMS), and matrix. (E) Stacked bar plots show fraction MS intensity for all *B. divergens* proteins detected (n = 3,414), colored by *T. gondii* homolog assigned to 12 subcellular compartments by hyperLOPIT,^11^ otherwise labeled Unknown (gray). Colors shown in panel F. (F) Relative protein abundance of *B. divergens* proteins across the mitochondrial enrichment protocol is shown for 12 subcellular compartments based on homology to *T. gondii* with hyperLOPIT annotations,^11^ counts indicated in parentheses. (G) Relative protein abundance shown for three mitochondrial marker proteins (top) and all 3,414 *B. divergens* proteins detected by mass spectrometry, ordered by PCP score (Pearson correlation to markers). The MitoViaPCP set is defined as PCP score ≥ 0.96. (H) Sensitivity-precision curves of the PCP score and the fractions collected throughout the enrichment protocol, calculated as log_2_(fraction/whole cell). Related to Figure S1 and Data S2, S3, S4.

**Figure 2.**
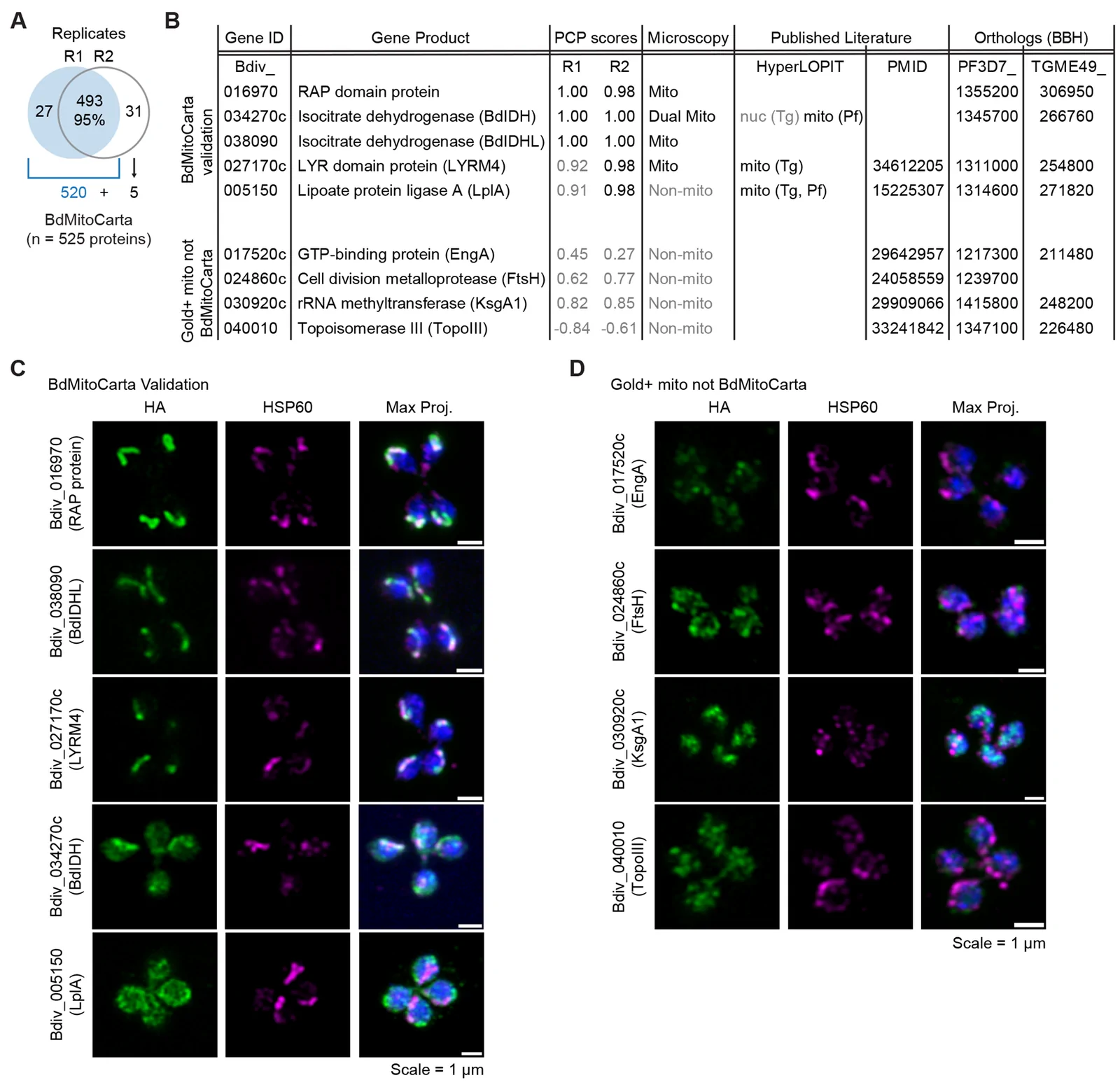
BdMitoCarta and microscopy validation. (A) Venn diagram shows overlap between two independent replicates of mitochondrial enrichment, DIA-MS, and PCP. Since replicate 2 showed higher FDR (18%), replicate 1 was used to define BdMitoCarta with the addition of 5 selected proteins from replicate 2 (see Methods). (B) Table shows five BdMitoCarta proteins selected for microscopy validation as well as four literature-based Gold+ mitochondrial proteins that scored below threshold 0.96 in our PCP. Gray font highlights evidence against mitochondrial localization (PCP score ≤ 0.96, or non-mitochondrial localization in hyperLOPIT or our microscopy). Prefixes for gene identifiers are shown in header (Bdiv_, PF3D7_, TGME49_). R1: Replicate 1; R2: Replicate 2. PMIDs are provided for proteins characterized in published literature.^58–63^ (C) Immunofluorescence microscopy analysis of five HA-tagged BdMitoCarta proteins. HSP60 was used as the mitochondrial marker. Scale 1 μm. (D) Immunofluorescence microscopy analysis of four HA-tagged Gold+ mito proteins that scored below threshold 0.96 in our PCP. HSP60 was used as the mitochondrial marker. Scale 1 μm. See Figure S3 for images of single and paired merozoites. Related to Figures S2, S3 and Data S3.

**Figure 3.**
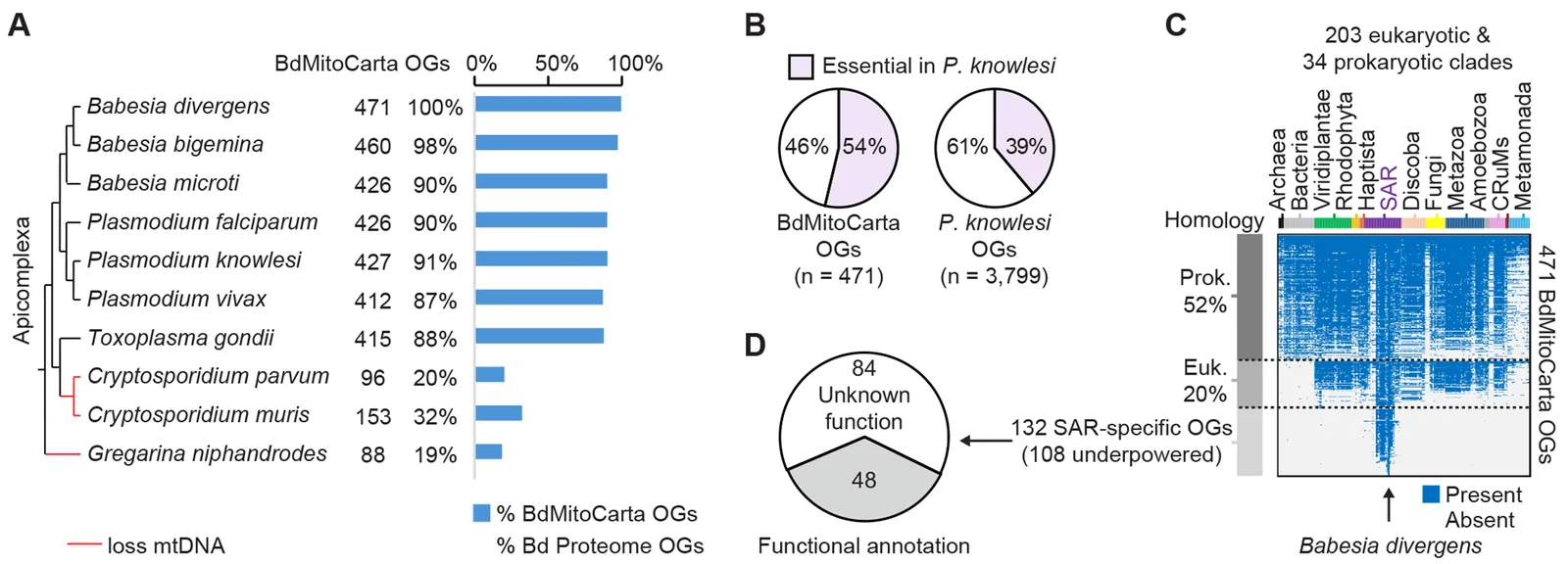
BdMitoCarta is highly conserved across Apicomplexa, enriched for essential genes, and contains many functionally uncharacterized proteins. (A) Bar plots for 10 Apicomplexan parasites show the fraction of 471 BdMitoCarta orthogroups (OGs, blue bars) with members in each species, as well as the fraction of all *B. divergens* proteome OGs (gray bars). Phylogenetic tree is adapted from Chen et al (co-submitted). We note that there is uncertainty with *Gregarina* placement. (B) Pie charts show the fraction of BdMitoCarta orthogroups that score as essential in *P. knowlesi* (hybrid model essentiality score < 0.26)^45^, compared to the similar metric for all *P. knowlesi* orthogroups. (C) Heatmap shows presence (blue) or absence (gray) of 471 BdMitoCarta orthogroups (rows) across 203 eukaryotic species and 34 prokaryotic clades (columns). Stacked bar on left shows homology to prokaryotic (Prok.) or eukaryotic (Euk.) members, as well as 132 OG with detectable members only in clade SAR, of which 108 are underpowered to detect homology based on abSENSE-HMM modeling (Chen et al, co-submitted). (D) Pie chart shows the proportion of SAR-specific OGs (n = 132) that have functional annotations from PFAM or PiroplasmaDB gene product descriptions. Related to Figure S4 and Data S3, S5.

**Figure 4.**
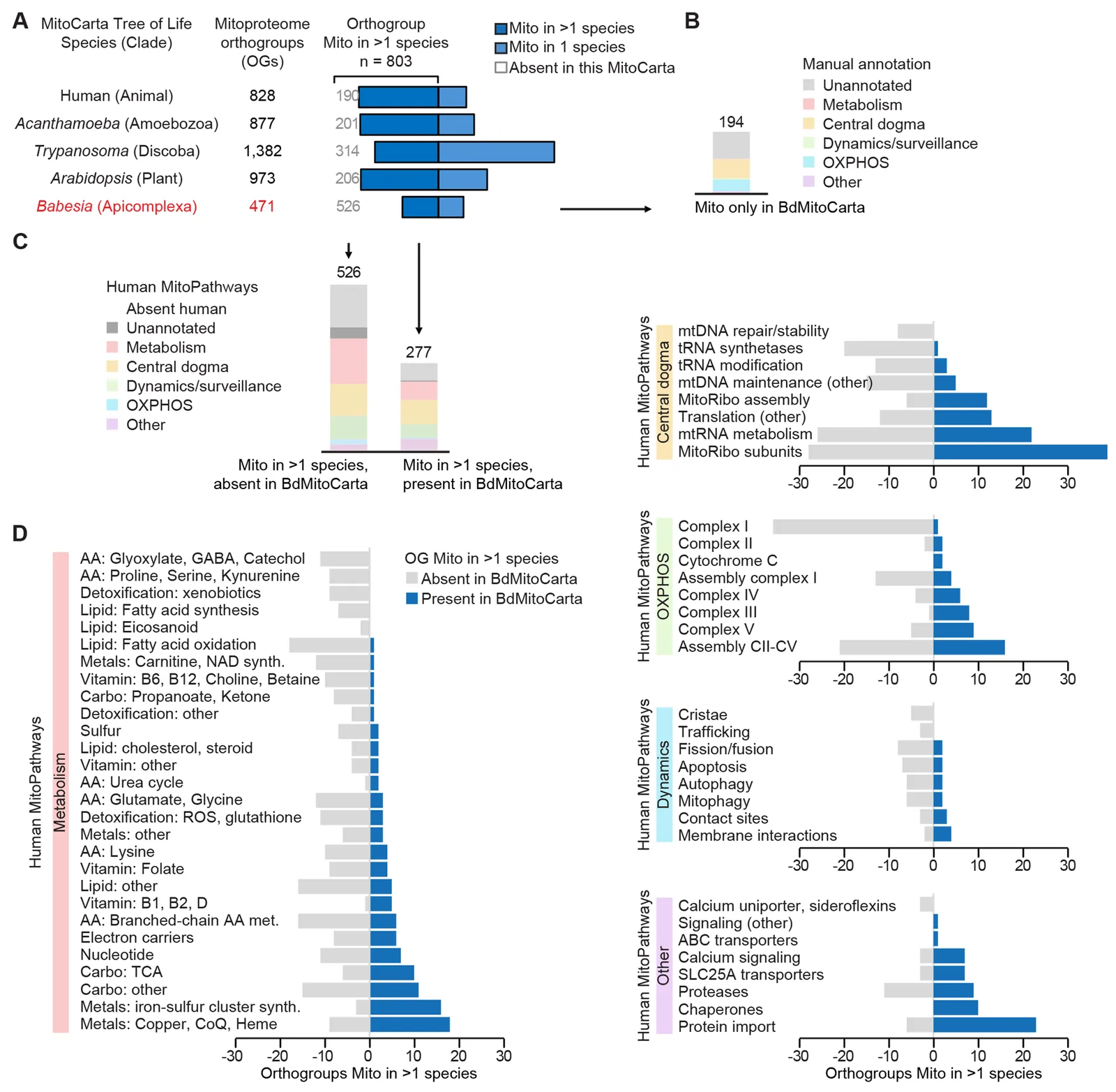
*B. divergens* mitoproteome is highly reduced compared to other characterized mitoproteomes. (A) Comparison of mitoproteomes across five highly divergent eukaryotic species in the Mitochondrial Tree of Life (MitoTOL) project, based on orthogroup (OG) homology. (B) Bar plot shows functional analysis of all 194 OGs with members in BdMitoCarta but not detected in the four other MitoCarta inventories. Manual annotations were based on a combination of *B. divergens* gene annotations, protein domains (PFAM), and homology to *P. falciparum or T. gondii* proteins with known functions (see Data S3). (C) Bar plots show OGs with members in >1 mitoproteome, and either absent or present in BdMitoCarta, colored by human MitoPathway annotation.^49^ (D) Bar plots show OGs with members in >1 mitoproteome and either absent (gray) or present (blue) in BdMitoCarta, grouped by human MitoPathway category. Abbreviations: AA: amino acid; Carbo: carbohydrate; synth: synthesis; met: metabolism; MitoRibo: mitochondrial ribosome. Related to Data S3, S5.

**Figure 5.**
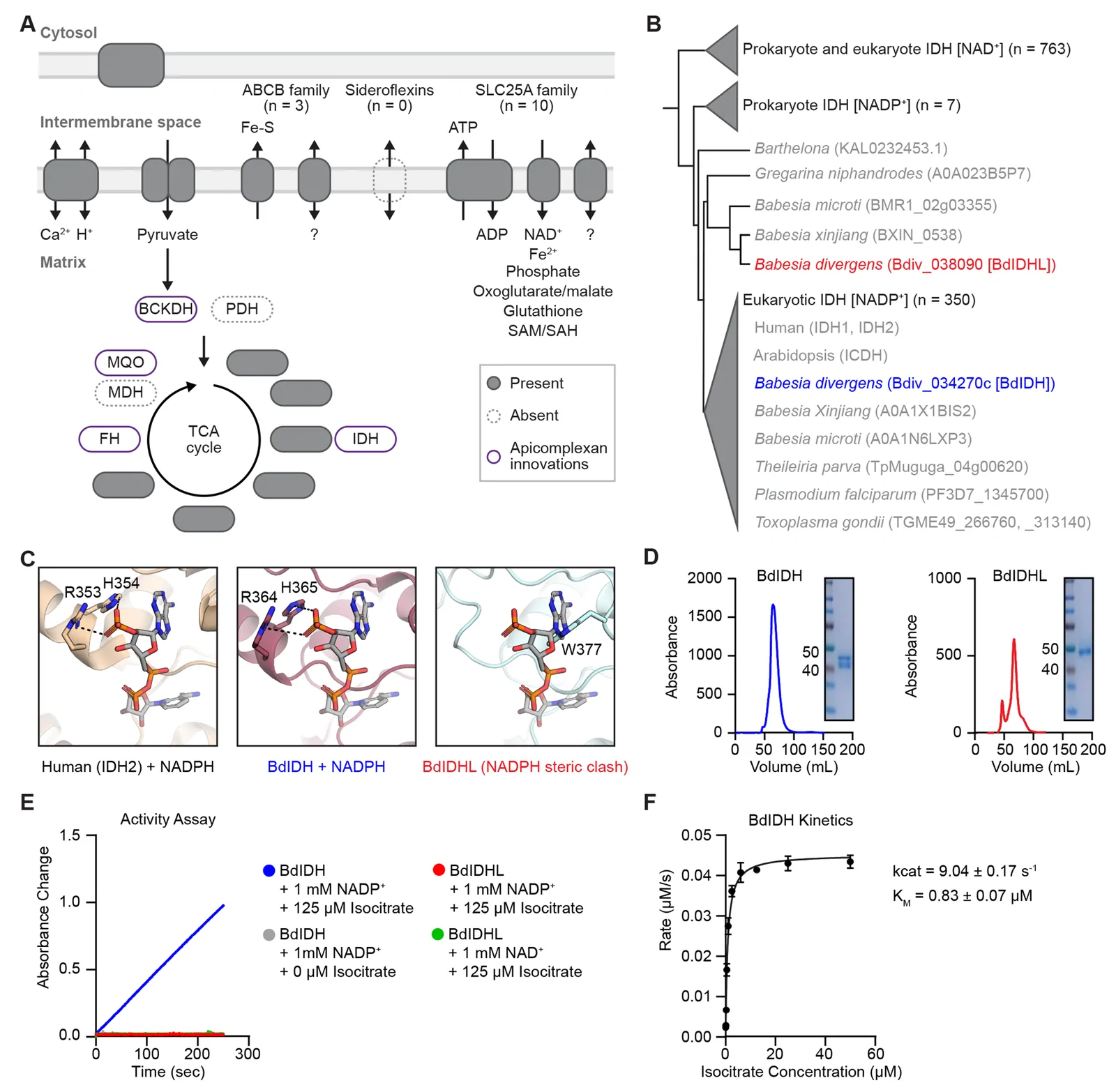
TCA cycle metabolism in *B. divergens*. (A) Diagram of the mitochondrial transporters and central carbon metabolism enzymes present in *B. divergens,* based on manual annotation of our BdMitoCarta inventory (see also Data S3). Human proteins that function in these processes but lack homologs in BdMitoCarta are denoted with dashed outlines. Apicomplexan-specific innovations (repurposed proteins or proteins not found in humans) are highlighted in purple. (B) Protein phylogeny depicting the evolutionary history of isocitrate dehydrogenase (IDH) enzymes, including the two IDH homologs found in the BdMitoCarta inventory. Clades collapsed for visualization. (C) Crystal structure of human mitochondrial NADP^+^-dependent IDH protein (IDH2) with bound NADPH (PDB 4JA8) and AlphaFold3^53^ predicted structures of the two *B. divergens* NADP^+^-dependent IDH protein homologs with NADPH docked into the NADP^+^ binding pocket through structural alignment with 4JA8. Key residues are rendered as sticks. (D) Gel filtration profile at 280 nm absorbance of final BdIDH and BdIDHL preparations and purity as demonstrated by SDS-PAGE. The elution volume of the main peak for both proteins corresponds to a homodimer. The double bands in the BdIDH gel correspond to full length and an N-terminal truncation of BdIDH as determined by mass spectrometry. (E) Activity of BdIDH and BdIDHL in the presence of isocitrate and either NADP^+^ or NAD_+_ as determined by the absorbance change at 340 nm upon reduction of NADP^+^/NAD^+^. (F) Steady-state enzyme kinetics of BdIDH as a function of increasing isocitrate concentration. Error bars indicate standard error of the mean from replicates in triplicate. Related to Figure S5 and Data S3.

**Figure 6.**
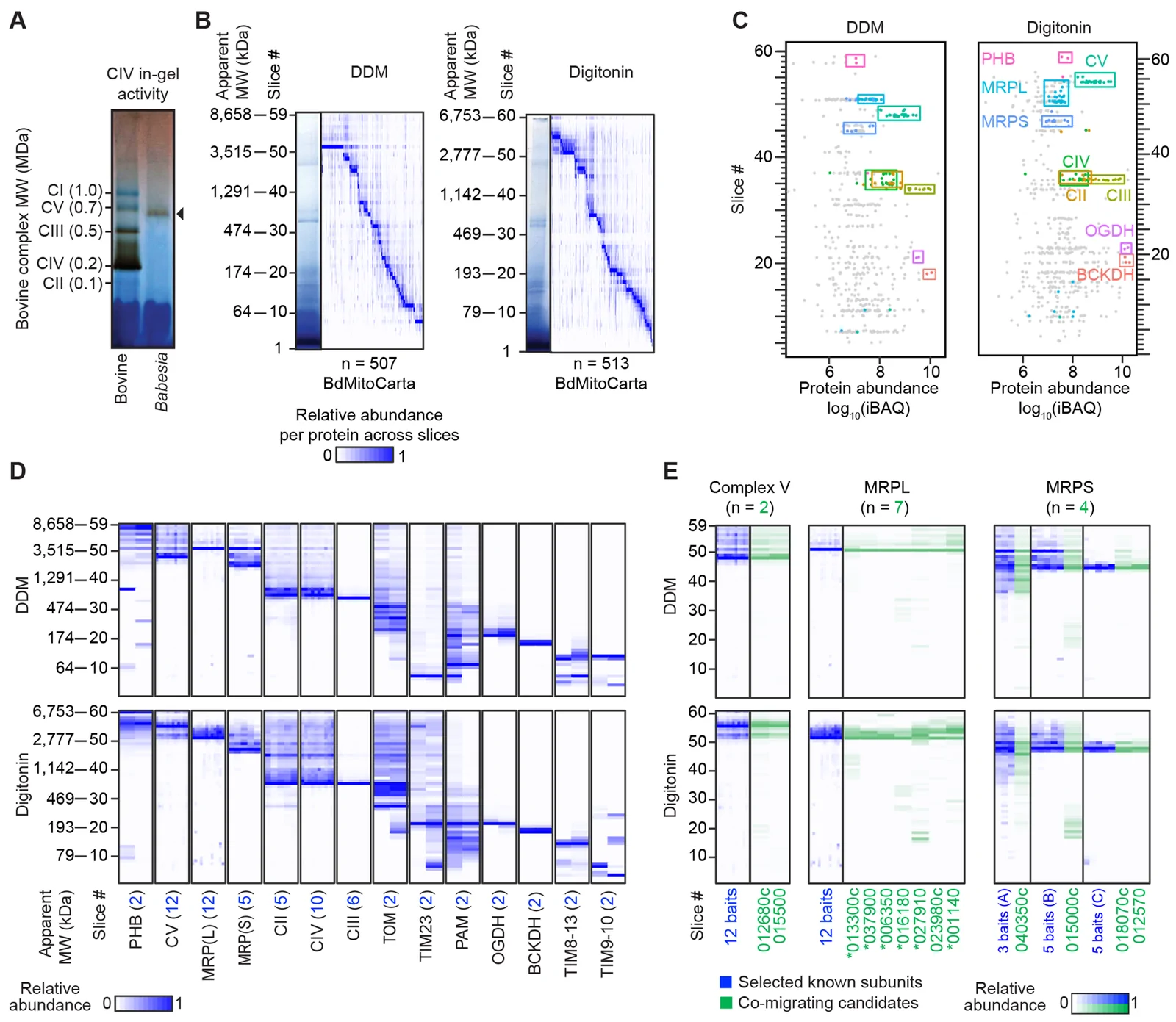
BN-PAGE complexome analysis assigns BdMitoCarta proteins to complexes. In-gel activity assay for OXPHOS complex IV (CIV). Triangle indicates primary enzyme activity band. (A) BN-PAGE gels cut into 60 slices each for MS analysis. Heatmaps indicate relative abundances of mitochondrial proteins detected, with each protein normalized to its maximum intensity across gel slices in each lane. Apparent MWs for slices are estimates based on migration of bovine OXPHOS complexes (Data S6). (B) Abundance of BdMitoCarta proteins identified in each BN-PAGE slice based on iBAQ MS proteomics shows that known complex members (colors) both co-migrate (Y-axis) and have similar abundance (X-axis). For visualization, only slices containing proteins with relative abundance ≥ 0.9 are displayed. (C) Heatmaps indicate relative abundances of strongly co-migrating protein members of each of the indicated complexes (parenthesis indicated number of selected subunits shown). Complexes are ordered by average slice of maximum intensity in digitonin. (D) Heatmaps show baits for selected complexes (blue) and co-migrating candidates (green) showing Pearson correlation ≥ 0.9 with mean bait profile across one or both detergents. Asterisk indicates homology to known ribosome assembly/binding domains. Related to Figures S6, S7, S8 and Data S6.

**Table:** Key resources table

REAGENT or RESOURCE	SOURCE	IDENTIFIER
Antibodies		
Rat monoclonal anti-HA antibody	Roche	Cat#11867423001
Guinea pig monoclonal anti-HA antibody	ABCD Antibodies	Cat#ABCD_AF291
Rabbit monoclonal anti-HA antibody	Cell Signaling	Cat#3724S
Rabbit polyclonal anti-HSP60 antibody	Novus Biologicals	Cat#NBP2-12734
Mouse monoclonal anti-Cytochrome c antibody	Abcam	Cat#ab13575
Rabbit polyclonal anti-LDH antibody	LSBIO	Cat#LS-C488831
Alexa Fluor 488 goat anti-rat antibody	Invitrogen	Cat#A-11001
Alexa Fluor 555 goat anti-rabbit antibody	Invitrogen	Cat#A-21428
Alexa Fluor 488 goat anti-guinea pig antibody	Invitrogen	Cat#A-11073
Alexa Fluor 405 NHS Ester	Invitrogen	Cat#A30000
Anti-rabbit IgG HRP-linked antibody	Cell Signaling	Cat#7074S
Anti-mouse IgG HRP-linked antibody	Sigma	Cat#GENA931
Chemicals, peptides, and recombinant proteins		
Hoescht	Thermo	Cat#62249
MitoTracker Orange CMTMRos	Thermo	Cat#M7510
Sytox Deep Red	Invitrogen	Cat#S11381
VectaShield Vibrance Mounting Media	Vector Laboratories	Cat#H-1700
OptiPrep density gradient medium	Sigma	Cat#D1556
cOmplete^™^ Protease Inhibitor Cocktail	Roche	Cat#65741000
Aprotinin	Sigma	Cat#A6106-1MG
Leupeptin	Roche	Cat#11017101001
Sequencing grade modified trypsin	Promega	Cat#V5113
Lysyl EndopeptidaseR, mass spectrometry grade (Lys-C)	FUJIFILM Wako Pure Chemical Corporation	Cat#129-02541
Deposited data		
*B. divergens* mitochondrial protein correlation profiling (MitoPCP) mass spectrometry proteomics dataset	This paper	massive.ucsd.edu: MSV000102179
*B. divergens* Blue Native PAGE Mass Spectrometry (BN-PAGE-MS) proteomics dataset	This paper	massive.ucsd.edu: MSV000102179
Experimental models: Cell lines		
Purified O+ human erythrocytes	Research Blood Components	N/A
Experimental models: Organisms/strains		
*Babesia divergens* strain Rouen 1987	Kirk Deitsch and Laura Kirkman (Weill Cornell Medical College)	N/A
Recombinant DNA		
pBE13 ectopic circular DNA plasmid for *B. divergens*	Brendan Elsworth (Harvard T.H. Chain School of Public Health)	N/A
0mod-prime_pBE13 ectopic circular DNA plasmid for *B. divergens*	This paper	N/A
pET-28A bacterial expression plasmid	EMD Biosciences	Addgene Plasmid #69864-3
Plasmid: 3xHA-eGFP-BdTOM22 in pBE13	This paper	N/A
Plasmid: 3xHA-eGFP in pBE13	This paper	N/A
Plasmid: 3xHA-Bdiv_016970 in 0mod-prime_pBE13	This paper	N/A
Plasmid: 3xHA- Bdiv_034270c in 0mod-prime_pBE13	This paper	N/A
Plasmid: 3xHA- Bdiv_027170c in 0mod-prime_pBE13	This paper	N/A
Plasmid: 3xHA- Bdiv_038090 in 0mod-prime_pBE13	This paper	N/A
Plasmid: 3xHA- Bdiv_005150 in 0mod-prime_pBE13	This paper	N/A
Plasmid: 3xHA- Bdiv_017520c in 0mod-prime_pBE13	This paper	N/A
Plasmid: 3xHA- Bdiv_024860c in 0mod-prime_pBE13	This paper	N/A
Plasmid: 3xHA- Bdiv_030920c in 0mod-prime_pBE13	This paper	N/A
Plasmid: 3xHA- Bdiv_040010 in 0mod-prime_pBE13	This paper	N/A
Plasmid: Bdiv_034270c-GGS-8xHIS in pET-28A	This paper	N/A
Plasmid: Bdiv_038090-GGS-8xHIS in pET-28A	This paper	N/A
Software and algorithms		
ZEISS ZEN (blue edition) 3.5	Carl Ziess Microscopy	www.zeiss.com
FIJI (Image J)	Schindelin et al.^65^	imagej.net
GraphPad Prism 11 (version 11.02.1)	GraphPad Software	www.graphpad.com
Spectronaut (version 19)	Biognosys	biognosys.com/software/spectronaut/
HMMER (version 3.1)	Potter et al.^68^	hmmer.org
OrthoFinder (version 2.5.5)	Emms et al.^71^	github.com/davidemms/OrthoFinder/releases
muscle5 (version 5.1)	Edgar et al.^72^	github.com/rcedgar/muscle/releases
ClipKIT (version 2.3.0)	Steenwyk et al.^73^	pypi.org/project/clipkit/
IQ-TREE (version 2.3.6)	Minh et al.^74^	github.com/iqtree/iqtree2/releases
AlphaFold3	Abramson et al.^53^	alphafoldserver.com
PyMOL	Schrödinger	www.pymol.org
Spectrum Mill	Broad Institute	proteomics.broadinstitute.org/millhome.htm
BLAST+ (version 2.2.30)	Camacho et al.^67^	ftp.ncbi.nlm.nih.gov/blast/executables/blast+/2.2.30/
Other		
Amaxa 4D-Nucleofector X Unit	Lonza	Cat#AAF-1003X
P3 Primary Cell 4D-Nucleofection Kit	Lonza	Cat#V4XP-3024
Ziess LSM-900 confocal microscope equipped with Airyscan 2	Carl Ziess Microscopy	N/A
TetraSpeck microspheres	Invitrogen	Cat#T14792
25 mm Swinnex filter holders	Sigma	Cat#SX0002500
2.0 μm pore size, 25 mm diameter polycarbonate membrane filters	Sigma	Cat#TTTP02500
μMACS^™^ anti-GFP microbeads	Miltenyi	Cat#130-091-125
Cell disruption vessel	Parr Instrument Company	Cat#4639
30 μm pre-separation filter	Miltenyi	Cat#130-041-407
LS columns	Miltenyi	Cat#130-042-401
QuadroMACS^™^ magnetic separator stand	Miltenyi	Cat#130-091-051
14 x 89 mm thinwall polypropylene tubes	Beckman/Fisher	Cat#NC9336079
SW 41 Ti swinging bucket rotor	Beckman	Cat#333790
S-Trap Mini Column	PROTIFI	Cat#C02-mini-40
S-Trap Micro Column	PROTIFI	Cat#C02-micro-40
Empore C18 Extraction Disks	CSD Analytical	Cat#76333-132
Evotip Pure, 96 tips	Evosep	Cat#EV2011
Endurance Column, 30 SPD	Evosep	Cat#EV1106
Stainless steel emitters	Evosep	Cat#EV1086
96-well microplate flat bottom clear	VWR	Cat#82050-760
PepSep C18 column (25 cm x 75 μm, 1.5 μm)	Bruker Daltonics	Cat#1893484
Endurance Column (15 cm × 0.15 mm, 1.9 μm)	Evosep Biosystems	Cat#EV1106
UNIPLATE microplates	Whatman	Cat#7701-5200
Sep-Pak tC18 96-well plate, 40 mg sorbent per well	Waters	Cat#186002320
Orbitrap Astral mass spectrometer	Thermo	N/A
Vanquish Neo UHPLC system	Thermo	N/A
Q Exactive^™^ Plus Hybrid Quadrupole-Orbitrap^™^ Mass Spectrometer	Thermo	N/A
Talon cobalt resin	Takara	Cat#635503
HiLoad 16/600 Superdex 200 prep grade column	Cytiva	Cat#28989335
NativePAGE Sample Prep Kit	Thermo	Cat#BN2008
Bovine heart mitochondria	Abcam	Cat#110338
NativePAGE Novex 3% to 12% Bis-Tris protein gel	Thermo	Cat#BN2011BX10
NativePAGE Running Buffer Kit	Thermo	Cat#BN2007
NativeMark Unstained Protein Standard	Thermo	Cat#LC0725

## Data Availability

BdMitoCarta is available at mitocarta.org. The original mass spectra and the protein sequence databases used for searches have been deposited in the public proteomics repository MassIVE (http://massive.ucsd.edu) and are accessible at ftp://MSV000102179@massive-ftp.ucsd.edu when providing the dataset password: babesia. These datasets will be made public upon acceptance of the manuscript. Processed proteomic data are available as supplemental tables. Code for the orthogroup inference and HGT analysis pipeline will be made available on GitHub: https://github.com/michaelzhuchen. Any additional information required to reanalyze the data reported in this paper is available from the senior corresponding authors (Vamsi K. Mootha vamsi@hms.harvard.edu or Manoj T. Duraisingh, mduraisi@hsph.harvard.edu) upon request.

## HIGHLIGHTS

- Experimental workflow enriches mitochondria from the intracellular parasites
- Protein correlation profiling defines the BdMitoCarta inventory with 525 proteins
- Identification of two mitochondrial isocitrate dehydrogenase homologs
- 14 mitochondrial complexes mapped by Blue Native PAGE mass spectrometry

## References

[1] Eme L, Sharpe SC, Brown MW, and Roger AJ. (2014). On the age of eukaryotes: evaluating evidence from fossils and molecular clocks. Cold Spring Harb Perspect Biol 6. 10.1101/cshperspect.a016139. PMC4107988 25085908

[2] Piro F, Focaia R, Dou Z, Masci S, Smith D, and Di Cristina M. (2021). An Uninvited Seat at the Dinner Table: How Apicomplexan Parasites Scavenge Nutrients from the Host. Microorganisms 9. 10.3390/microorganisms9122592. PMC8707601 34946193

[3] Wasmuth J, Daub J, Peregrin-Alvarez JM, Finney CA, and Parkinson J. (2009). The origins of apicomplexan sequence innovation. Genome Res 19, 1202–1213. 10.1101/gr.083386.108. PMC2704437 19363216

[4] Burki F, Roger AJ, Brown MW, and Simpson AGB. (2020). The New Tree of Eukaryotes. Trends Ecol Evol 35, 43–55. 10.1016/j.tree.2019.08.008. 31606140

[5] Lamb IM, Okoye IC, Mather MW, and Vaidya AB. (2023). Unique Properties of Apicomplexan Mitochondria. Annu Rev Microbiol 77, 541–560. 10.1146/annurev-micro-032421-120540. PMC11156254 37406344

[6] Mathur V, Wakeman KC, and Keeling PJ. (2021). Parallel functional reduction in the mitochondria of apicomplexan parasites. Curr Biol 31, 2920–2928 e2924. 10.1016/j.cub.2021.04.028. 33974849

[7] Berna L, Rego N, and Francia ME. (2021). The Elusive Mitochondrial Genomes of Apicomplexa: Where Are We Now? Front Microbiol 12, 751775. 10.3389/fmicb.2021.751775. PMC8554336 34721355

[8] Wilson RJ, and Williamson DH. (1997). Extrachromosomal DNA in the Apicomplexa. Microbiol Mol Biol Rev 61, 1–16. 10.1128/mmbr.61.1.1-16.1997. PMC232597 9106361

[9] Liu S, Roellig DM, Guo Y, Li N, Frace MA, Tang K, Zhang L, Feng Y, and Xiao L. (2016). Evolution of mitosome metabolism and invasion-related proteins in Cryptosporidium. BMC Genomics 17, 1006. 10.1186/s12864-016-3343-5. PMC5146892 27931183

[10] Guerin A, Strelau KM, Barylyuk K, Wallbank BA, Berry L, Crook OM, Lilley KS, Waller RF, and Striepen B. (2023). Cryptosporidium uses multiple distinct secretory organelles to interact with and modify its host cell. Cell Host Microbe 31, 650–664 e656. 10.1016/j.chom.2023.03.001. 36958336

[11] Barylyuk K, Koreny L, Ke H, Butterworth S, Crook OM, Lassadi I, Gupta V, Tromer E, Mourier T, Stevens TJ, (2020). A Comprehensive Subcellular Atlas of the Toxoplasma Proteome via hyperLOPIT Provides Spatial Context for Protein Functions. Cell Host Microbe 28, 752–766 e759. 10.1016/j.chom.2020.09.011. PMC7670262 33053376

[12] Chen M, Koszti SG, Bonavoglia A, Maco B, von Rohr O, Peng HJ, Soldati-Favre D, and Kloehn J. (2025). Dissecting apicoplast functions through continuous cultivation of Toxoplasma gondii devoid of the organelle. Nat Commun 16, 2095. 10.1038/s41467-025-57302-x. PMC11873192 40025025

[13] Denton H, Roberts CW, Alexander J, Thong KW, and Coombs GH. (1996). Enzymes of energy metabolism in the bradyzoites and tachyzoites of Toxoplasma gondii. FEMS Microbiol Lett 137, 103–108. 10.1111/j.1574-6968.1996.tb08090.x. 8935663

[14] Fleige T, Limenitakis J, and Soldati-Favre D. (2010). Apicoplast: keep it or leave it. Microbes Infect 12, 253–262. 10.1016/j.micinf.2009.12.010. 20083219

[15] He TY, Li YT, Liu ZD, Cheng H, Bao YF, and Zhang JL. (2024). Lipid metabolism: the potential targets for toxoplasmosis treatment. Parasit Vectors 17, 111. 10.1186/s13071-024-06213-9. PMC10916224 38448975

[16] Sun SY, Segev-Zarko L.a., Pintilie GD, Kim CY, Staggers SR, Schmid MF, Egan ES, Chiu W, and Boothroyd JC. (2024). Cryogenic electron tomography reveals novel structures in the apical complex of Plasmodium falciparum. mBio 15, e0286423. 10.1128/mbio.02864-23. PMC11005440 38456679

[17] Blauwkamp JA, Rajaram K, Staggers SR, Harrigan O, Doud EH, Xu W, Ke H, Prigge ST, Sun SY, and Absalon S. (2025). An essential adaptor for apicoplast fission and inheritance in malaria parasites. Nat Commun 16, 11325. 10.1038/s41467-025-66393-5. PMC12722261 41309594

[18] Chisholm SA, Flores V, Kemp A, Breckels LM, Koreny L, Dos Santos Pacheco N, Barylyuk K, Kuroshchenkova A, Lilley KS, Rayner JC, and Waller RF. (2026). The spatial proteome of the Plasmodium falciparum schizont illuminates the composition and evolutionary trajectories of its organelles. Nat Commun. 10.1038/s41467-026-73664-2. PMC13369866 42218142

[19] van Esveld SL, Meerstein-Kessel L, Boshoven C, Baaij JF, Barylyuk K, Coolen JPM, van Strien J, Duim RAJ, Dutilh BE, Garza DR, (2021). A Prioritized and Validated Resource of Mitochondrial Proteins in Plasmodium Identifies Unique Biology. mSphere 6, e0061421. 10.1128/mSphere.00614-21. PMC8550323 34494883

[20] Seidi A, Muellner-Wong LS, Rajendran E, Tjhin ET, Dagley LF, Aw VY, Faou P, Webb AI, Tonkin CJ, and van Dooren GG. (2018). Elucidating the mitochondrial proteome of Toxoplasma gondii reveals the presence of a divergent cytochrome c oxidase. Elife 7. 10.7554/eLife.38131. PMC6156079 30204084

[21] Lamb IM, Rios KT, Shukla A, Ahiya AI, Morrisey J, Mell JC, Lindner SE, Mather MW, and Vaidya AB. (2022). Mitochondrially targeted proximity biotinylation and proteomic analysis in Plasmodium falciparum. PLoS One 17, e0273357. 10.1371/journal.pone.0273357. PMC9390924 35984838

[22] Evers F, Cabrera-Orefice A, Elurbe DM, Kea-Te Lindert M, Boltryk SD, Voss TS, Huynen MA, Brandt U, and Kooij TWA. (2021). Composition and stage dynamics of mitochondrial complexes in Plasmodium falciparum. Nat Commun 12, 3820. 10.1038/s41467-021-23919-x. PMC8217502 34155201

[23] Maclean AE, Bridges HR, Silva MF, Ding S, Ovciarikova J, Hirst J, and Sheiner L. (2021). Complexome profile of Toxoplasma gondii mitochondria identifies divergent subunits of respiratory chain complexes including new subunits of cytochrome bc1 complex. PLoS Pathog 17, e1009301. 10.1371/journal.ppat.1009301. PMC7987180 33651838

[24] Homer MJ, Aguilar-Delfin I, Telford SR 3rd, Krause PJ, and Persing DH. (2000). Babesiosis. Clin Microbiol Rev 13, 451–469. 10.1128/CMR.13.3.451. PMC88943 10885987

[25] Hunfeld KP, Hildebrandt A, and Gray JS. (2008). Babesiosis: recent insights into an ancient disease. Int J Parasitol 38, 1219–1237. 10.1016/j.ijpara.2008.03.001. 18440005

[26] Laha R, Das M, and Sen A. (2015). Morphology, epidemiology, and phylogeny of Babesia: An overview. Trop Parasitol 5, 94–100. 10.4103/2229-5070.162490. PMC4557164 26629451

[27] Vannier E, Gewurz BE, and Krause PJ. (2008). Human babesiosis. Infect Dis Clin North Am 22, 469–488, viii-ix. 10.1016/j.idc.2008.03.010. PMC3998201 18755385

[28] Jalovecka M, Sojka D, Ascencio M, and Schnittger L. (2019). Babesia Life Cycle - When Phylogeny Meets Biology. Trends Parasitol 35, 356–368. 10.1016/j.pt.2019.01.007. 30733093

[29] Jacob SS, Sengupta PP, Paramanandham K, Suresh KP, Chamuah JK, Rudramurthy GR, and Roy P. (2020). Bovine babesiosis: An insight into the global perspective on the disease distribution by systematic review and meta-analysis. Vet Parasitol 283, 109136. 10.1016/j.vetpar.2020.109136. 32574977

[30] Kumar A, O’Bryan J, and Krause PJ. (2021). The Global Emergence of Human Babesiosis. Pathogens 10. 10.3390/pathogens10111447. PMC8623124 34832603

[31] Krause PJ, Lepore T, Sikand VK, Gadbaw J Jr., Burke G, Telford SR 3rd, Brassard P, Pearl D, Azlanzadeh J, Christianson D, (2000). Atovaquone and azithromycin for the treatment of babesiosis. N Engl J Med 343, 1454–1458. 10.1056/NEJM200011163432004. 11078770

[32] Renard I, and Ben Mamoun C. (2021). Treatment of Human Babesiosis: Then and Now. Pathogens 10. 10.3390/pathogens10091120. PMC8469882 34578153

[33] Hikosaka K, Watanabe Y, Tsuji N, Kita K, Kishine H, Arisue N, Palacpac NM, Kawazu S, Sawai H, Horii T, (2010). Divergence of the mitochondrial genome structure in the apicomplexan parasites, Babesia and Theileria. Mol Biol Evol 27, 1107–1116. 10.1093/molbev/msp320. 20034997

[34] Brayton KA, Lau AO, Herndon DR, Hannick L, Kappmeyer LS, Berens SJ, Bidwell SL, Brown WC, Crabtree J, Fadrosh D, (2007). Genome sequence of Babesia bovis and comparative analysis of apicomplexan hemoprotozoa. PLoS Pathog 3, 1401–1413. 10.1371/journal.ppat.0030148. PMC2034396 17953480

[35] Liu Q, Guan XA, Li DF, Zheng YX, Wang S, Xuan XN, Zhao JL, and He L. (2023). Babesia gibsoni Whole-Genome Sequencing, Assembling, Annotation, and Comparative Analysis. Microbiol Spectr 11, e0072123. 10.1128/spectrum.00721-23. PMC10434002 37432130

[36] Macasev D, Whelan J, Newbigin E, Silva-Filho MC, Mulhern TD, and Lithgow T. (2004). Tom22’, an 8-kDa trans-site receptor in plants and protozoans, is a conserved feature of the TOM complex that appeared early in the evolution of eukaryotes. Mol Biol Evol 21, 1557–1564. 10.1093/molbev/msh166. 15155803

[37] Su J, Liu D, Yang F, Zuo MQ, Li C, Dong MQ, Sun S, and Sui SF. (2022). Structural basis of Tom20 and Tom22 cytosolic domains as the human TOM complex receptors. Proc Natl Acad Sci U S A 119, e2200158119. 10.1073/pnas.2200158119. PMC9245660 35733257

[38] Hornig-Do HT, Gunther G, Bust M, Lehnartz P, Bosio A, and Wiesner RJ. (2009). Isolation of functional pure mitochondria by superparamagnetic microbeads. Anal Biochem 389, 1–5. 10.1016/j.ab.2009.02.040. 19285029

[39] Guzman UH, Martinez-Val A, Ye Z, Damoc E, Arrey TN, Pashkova A, Renuse S, Denisov E, Petzoldt J, Peterson AC, (2024). Ultra-fast label-free quantification and comprehensive proteome coverage with narrow-window data-independent acquisition. Nat Biotechnol 42, 1855–1866. 10.1038/s41587-023-02099-7. PMC11631760 38302753

[40] Johnson WC, Taus NS, Reif KE, Bohaliga GA, Kappmeyer LS, and Ueti MW. (2017). Analysis of Stage-Specific Protein Expression during Babesia Bovis Development within Female Rhipicephalus Microplus. J Proteome Res 16, 1327–1338. 10.1021/acs.jproteome.6b00947. 28152313

[41] Foster LJ, de Hoog CL, Zhang Y, Zhang Y, Xie X, Mootha VK, and Mann M. (2006). A mammalian organelle map by protein correlation profiling. Cell 125, 187–199. 10.1016/j.cell.2006.03.022. 16615899

[42] Fukasawa Y, Oda T, Tomii K, and Imai K. (2017). Origin and Evolutionary Alteration of the Mitochondrial Import System in Eukaryotic Lineages. Mol Biol Evol 34, 1574–1586. 10.1093/molbev/msx096. PMC5455965 28369657

[43] Garcia PS, D’Angelo F, Ollagnier de Choudens S, Dussouchaud M, Bouveret E, Gribaldo S, and Barras F. (2022). An early origin of iron-sulfur cluster biosynthesis machineries before Earth oxygenation. Nat Ecol Evol 6, 1564–1572. 10.1038/s41559-022-01857-1. 36109654

[44] Rebeaud ME, Mallik S, Goloubinoff P, and Tawfik DS. (2021). On the evolution of chaperones and cochaperones and the expansion of proteomes across the Tree of Life. Proc Natl Acad Sci U S A 118. 10.1073/pnas.2020885118. PMC8166112 34001607

[45] Elsworth B, Ye S, Dass S, Tennessen JA, Sultana Q, Thommen BT, Paul AS, Kanjee U, Gruring C, Ferreira MU, (2025). The essential genome of Plasmodium knowlesi reveals determinants of antimalarial susceptibility. Science 387, eadq6241. 10.1126/science.adq6241. PMC12104972 39913579

[46] Maclean AE, Hayward JA, Huet D, van Dooren GG, and Sheiner L. (2022). The mystery of massive mitochondrial complexes: the apicomplexan respiratory chain. Trends Parasitol 38, 1041–1052. 10.1016/j.pt.2022.09.008. PMC10434753 36302692

[47] Mogi T, and Kita K. (2009). Identification of mitochondrial Complex II subunits SDH3 and SDH4 and ATP synthase subunits a and b in Plasmodium spp. Mitochondrion 9, 443–453. 10.1016/j.mito.2009.08.004. 19682605

[48] Muhleip A, Kock Flygaard R, Ovciarikova J, Lacombe A, Fernandes P, Sheiner L, and Amunts A. (2021). ATP synthase hexamer assemblies shape cristae of Toxoplasma mitochondria. Nat Commun 12, 120. 10.1038/s41467-020-20381-z. PMC7785744 33402698

[49] Rath S, Sharma R, Gupta R, Ast T, Chan C, Durham TJ, Goodman RP, Grabarek Z, Haas ME, Hung WHW, (2021). MitoCarta3.0: an updated mitochondrial proteome now with sub-organelle localization and pathway annotations. Nucleic Acids Res 49, D1541–D1547. 10.1093/nar/gkaa1011. PMC7778944 33174596

[50] Esseiva AC, Naguleswaran A, Hemphill A, and Schneider A. (2004). Mitochondrial tRNA import in Toxoplasma gondii. J Biol Chem 279, 42363–42368. 10.1074/jbc.M404519200. 15280394

[51] Oppenheim RD, Creek DJ, Macrae JI, Modrzynska KK, Pino P, Limenitakis J, Polonais V, Seeber F, Barrett MP, Billker O, (2014). BCKDH: the missing link in apicomplexan mitochondrial metabolism is required for full virulence of Toxoplasma gondii and Plasmodium berghei. PLoS Pathog 10, e1004263. 10.1371/journal.ppat.1004263. PMC4102578 25032958

[52] Pan K, Zeng A, Ruan X, Mo X, Shen B, Zhao J, and Zhou Y. (2025). The apicoplast localized isocitrate dehydrogenase is needed for de novo fatty acid synthesis in the apicoplast of Toxoplasma gondii. Front Cell Infect Microbiol 15, 1542122. 10.3389/fcimb.2025.1542122. PMC12234471 40630641

[53] Abramson J, Adler J, Dunger J, Evans R, Green T, Pritzel A, Ronneberger O, Willmore L, Ballard AJ, Bambrick J, (2024). Accurate structure prediction of biomolecular interactions with AlphaFold 3. Nature 630, 493–500. 10.1038/s41586-024-07487-w. PMC11168924 38718835

[54] Sun P, Liu Y, Ma T, and Ding J. (2020). Structure and allosteric regulation of human NAD-dependent isocitrate dehydrogenase. Cell Discov 6, 94. 10.1038/s41421-020-00220-7. PMC7752914 33349631

[55] Leonard RA, Tian Y, Tan F, van Dooren GG, and Hayward JA. (2023). An essential role for an Fe-S cluster protein in the cytochrome c oxidase complex of Toxoplasma parasites. PLoS Pathog 19, e1011430. 10.1371/journal.ppat.1011430. PMC10263302 37262100

[56] Shikha S, Tobiasson V, Ferreira Silva M, Ovciarikova J, Beraldi D, Muhleip A, and Sheiner L. (2025). Numerous rRNA molecules form the apicomplexan mitoribosome via repurposed protein and RNA elements. Nat Commun 16, 817. 10.1038/s41467-025-56057-9. PMC11742926 39827269

[57] Wang C, Kassem S, Rocha REO, Sun P, Nguyen TT, Kloehn J, Liu X, Brusini L, Bonavoglia A, Barua S, (2024). Apicomplexan mitoribosome from highly fragmented rRNAs to a functional machine. Nat Commun 15, 10689. 10.1038/s41467-024-55033-z. PMC11652630 39690155

[58] Falekun S, Sepulveda J, Jami-Alahmadi Y, Park H, Wohlschlegel JA, and Sigala PA. (2021). Divergent acyl carrier protein decouples mitochondrial Fe-S cluster biogenesis from fatty acid synthesis in malaria parasites. Elife 10. 10.7554/eLife.71636. PMC8547962 34612205

[59] Wrenger C, and Muller S. (2004). The human malaria parasite Plasmodium falciparum has distinct organelle-specific lipoylation pathways. Mol Microbiol 53, 103–113. 10.1111/j.1365-2958.2004.04112.x. 15225307

[60] Gupta K, Gupta A, Haider A, and Habib S. (2018). Characterization of mitochondrion-targeted GTPases in Plasmodium falciparum. Parasitology 145, 1600–1612. 10.1017/S0031182018000501. 29642957

[61] Tanveer A, Allen SM, Jackson KE, Charan M, Ralph SA, and Habib S. (2013). An FtsH protease is recruited to the mitochondrion of Plasmodium falciparum. PLoS One 8, e74408. 10.1371/journal.pone.0074408. PMC3772908 24058559

[62] Gupta K, Gupta A, and Habib S. (2018). Characterization of a Plasmodium falciparum rRNA methyltransferase. Mol Biochem Parasitol 223, 13–18. 10.1016/j.molbiopara.2018.06.001. 29909066

[63] Bansod S, Bung N, Singh P, Suthram N, Choudhury H, Roy A, Bulusu G, and Bhattacharyya S. (2020). Elucidation of an essential function of the unique charged domain of Plasmodium topoisomerase III. Biochem J. 10.1042/BCJ20200318. 33241842

[64] Sreenivasamurthy SK, Baptista CG, West CM, Blader IJ, and Dvorin JD. (2025). PfFBXO1 is essential for inner membrane complex formation in Plasmodium falciparum during both asexual and transmission stages. Commun Biol 8, 190. 10.1038/s42003-025-07619-6. PMC11802861 39915671

[65] Schindelin J, Arganda-Carreras I, Frise E, Kaynig V, Longair M, Pietzsch T, Preibisch S, Rueden C, Saalfeld S, Schmid B, (2012). Fiji: an open-source platform for biological-image analysis. Nat Methods 9, 676–682. 10.1038/nmeth.2019. PMC3855844 22743772

[66] Singh P, Vydyam P, Fang T, Estrada K, Gonzalez LM, Grande R, Kumar M, Chakravarty S, Berry V, Ranwez V, (2024). Insights into the evolution, virulence and speciation of Babesia MO1 and Babesia divergens through multiomics analyses. Emerg Microbes Infect 13, 2386136. 10.1080/22221751.2024.2386136. PMC11370697 39148308

[67] Camacho C, Coulouris G, Avagyan V, Ma N, Papadopoulos J, Bealer K, and Madden TL. (2009). BLAST+: architecture and applications. BMC Bioinformatics 10, 421. 10.1186/1471-2105-10-421. PMC2803857 20003500

[68] Potter SC, Luciani A, Eddy SR, Park Y, Lopez R, and Finn RD. (2018). HMMER web server: 2018 update. Nucleic Acids Res 46, W200–W204. 10.1093/nar/gky448. PMC6030962 29905871

[69] Odum MT, Teufel F, Thumuluri V, Almagro Armenteros JJ, Johansen AR, Winther O, and Nielsen H. (2024). DeepLoc 2.1: multi-label membrane protein type prediction using protein language models. Nucleic Acids Res 52, W215–W220. 10.1093/nar/gkae237. PMC11223819 38587188

[70] Calvo SE, Clauser KR, and Mootha VK. (2016). MitoCarta2.0: an updated inventory of mammalian mitochondrial proteins. Nucleic Acids Res 44, D1251–1257. 10.1093/nar/gkv1003. PMC4702768 26450961

[71] Emms DM, and Kelly S. (2019). OrthoFinder: phylogenetic orthology inference for comparative genomics. Genome Biol 20, 238. 10.1186/s13059-019-1832-y. PMC6857279 31727128

[72] Edgar RC. (2022). Muscle5: High-accuracy alignment ensembles enable unbiased assessments of sequence homology and phylogeny. Nat Commun 13, 6968. 10.1038/s41467-022-34630-w. PMC9664440 36379955

[73] Steenwyk JL, Buida TJ 3rd, Li Y, Shen XX, and Rokas A. (2020). ClipKIT: A multiple sequence alignment trimming software for accurate phylogenomic inference. PLoS Biol 18, e3001007. 10.1371/journal.pbio.3001007. PMC7735675 33264284

[74] Minh BQ, Schmidt HA, Chernomor O, Schrempf D, Woodhams MD, von Haeseler A, and Lanfear R. (2020). IQ-TREE 2: New Models and Efficient Methods for Phylogenetic Inference in the Genomic Era. Mol Biol Evol 37, 1530–1534. 10.1093/molbev/msaa015. PMC7182206 32011700

