# Supplementary material for: Mitochondrial proteome of the apicomplexan Babesia divergens
Source: Curr Biol. Author manuscript; Available in PMC 2026 Oct 17. (PMC13673260; doi:10.1016/j.cub.2026.08.072)
Supplement: Supplementary file 1 [file NIHMS2214436-supplement-1.pdf]

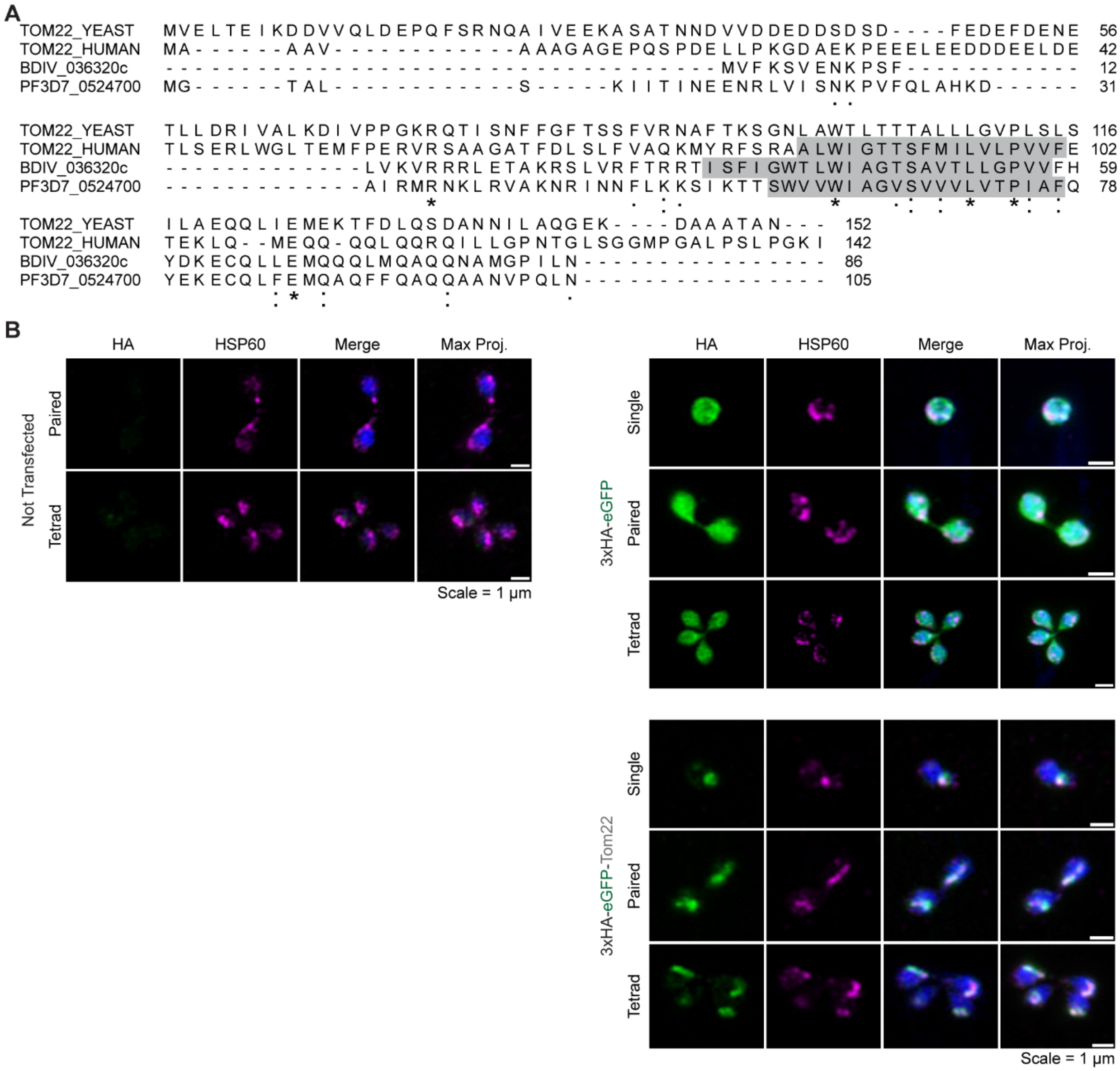

Figure S1. Additional MitoIP construct validation. Related to Figure 1.
(A) Multiple sequence alignments of yeast, human, *B. divergens,* and *P. falciparum* TOM22 proteins (Clustal Omega).[S1] Grey shading indicates transmembrane regions predicted by TMHMM. (.) indicates a semi-conserved residue; (:) indicates a strongly conserved residue; (*) indicates an identical residue. (B) Immunofluorescence microscopy analysis of MitoIP construct (3xHA-eGFP-BdTOM22) localization to the mitochondrion in single, paired, and tetrad *B. divergens* merozoites. Wildtype cells (not transfected) and cells expressing only the affinity tag (3xHA-eGFP) were analyzed as controls. HSP60 was used as the mitochondrial marker. Scale = 1 µm.

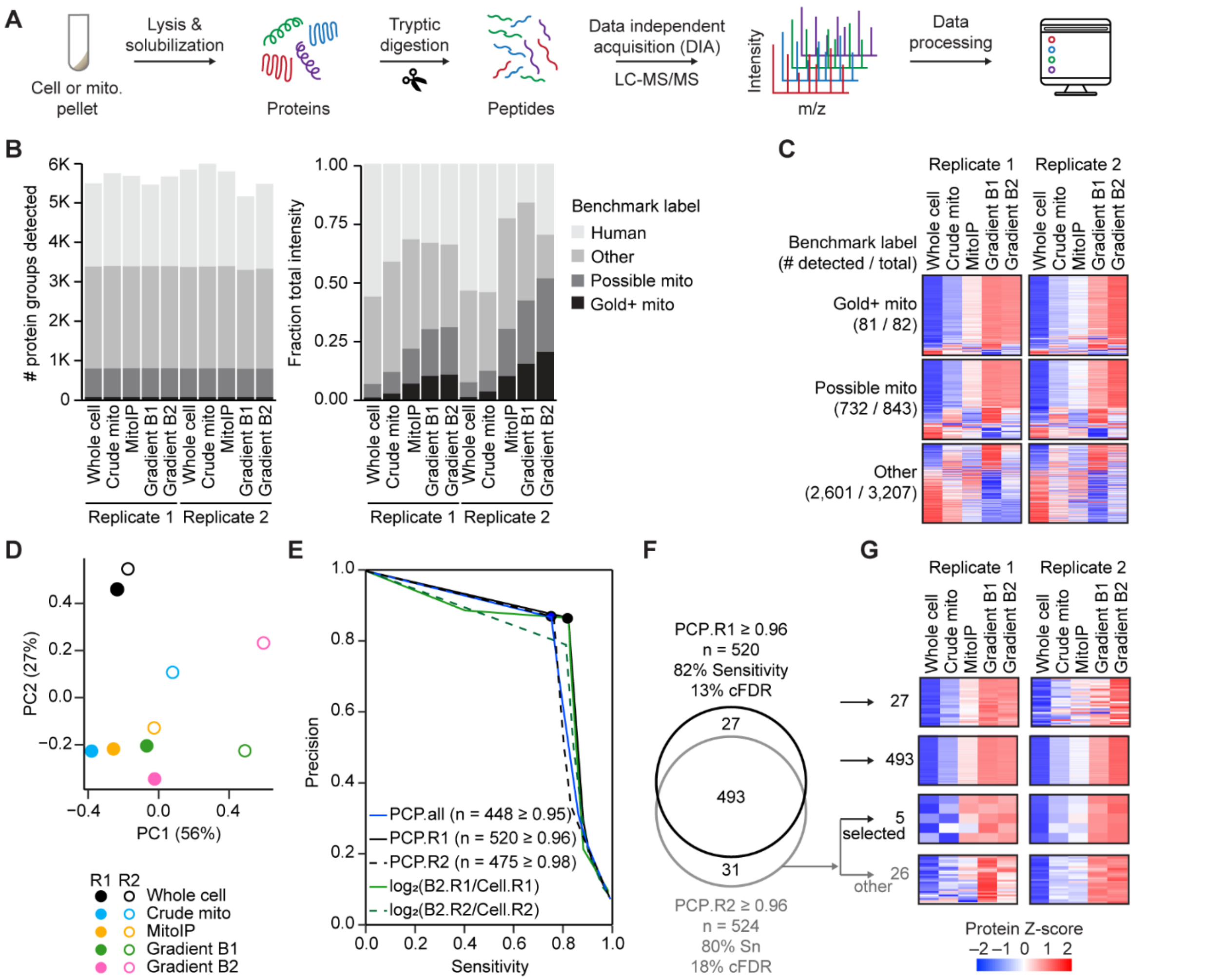

Figure S2. Mitochondrial enrichment protocol mass spectrometry results. Related to Figure 2 and Data S3, S4.

(A) Schematic of the mass spectrometry workflow for sample preparation and analysis using data independent acquisition mass spectrometry (DIA-MS). (B) Bar plots show the number and total MS intensity of *B. divergens* and human proteins detected in replicate 1 (R1) and replicate 2 (R2), performed six months apart. The *B. divergens* proteins are partitioned into the "gold+ mito", "possible mito", or "other" benchmark label categories. (C) Heatmaps show relative abundance for *B. divergens* proteins belonging to each benchmark label. Z-scores are calculated for each replicate separately, but gene order is preserved across replicates (ordered by PCP.R1). Color as in panel G. (D) Principal Component (PC) analysis for all samples shows substantial batch effects, with R2 (open circles) separating on PC1, motivating analysis of each replicate separately. Parentheses indicate % variance explained by each PC. (E) Sensitivity and precision based on benchmark data are shown for PCP scores calculated across all 10 samples (PCP.all) or limited to R1 (PCP.R1) or R2 (PCP.R2), with dots indicating MaxAUC (maximum Area Under Curve) correlation threshold (indicated in parentheses). Analysis shows that at 13% cFDR, PCP.R1 (82% sensitivity) outperforms PCP.all (75% sensitivity) and PCP.R2 (76% sensitivity). Thus, PCP.R1 was selected for creation of BdMitoCarta. (F) Venn diagram shows high overlap (493/520=95%) between PCP scores of PCP.R1 (black) and PCP.R2 (light gray) at the same correlation threshold of 0.96. (G) Heatmaps show relative protein abundance across the enrichment protocol for each section of the Venn diagram. Z-scores are calculated for each replicate separately, but gene order is preserved across replicates (ordered by PCP.R1). Of the 31 R2-only proteins, 5 were manually selected and added to BdMitoCarta (PCP.R2 > 0.98 and corroborating evidence from *T. gondii* hyperLOPIT).[S2]

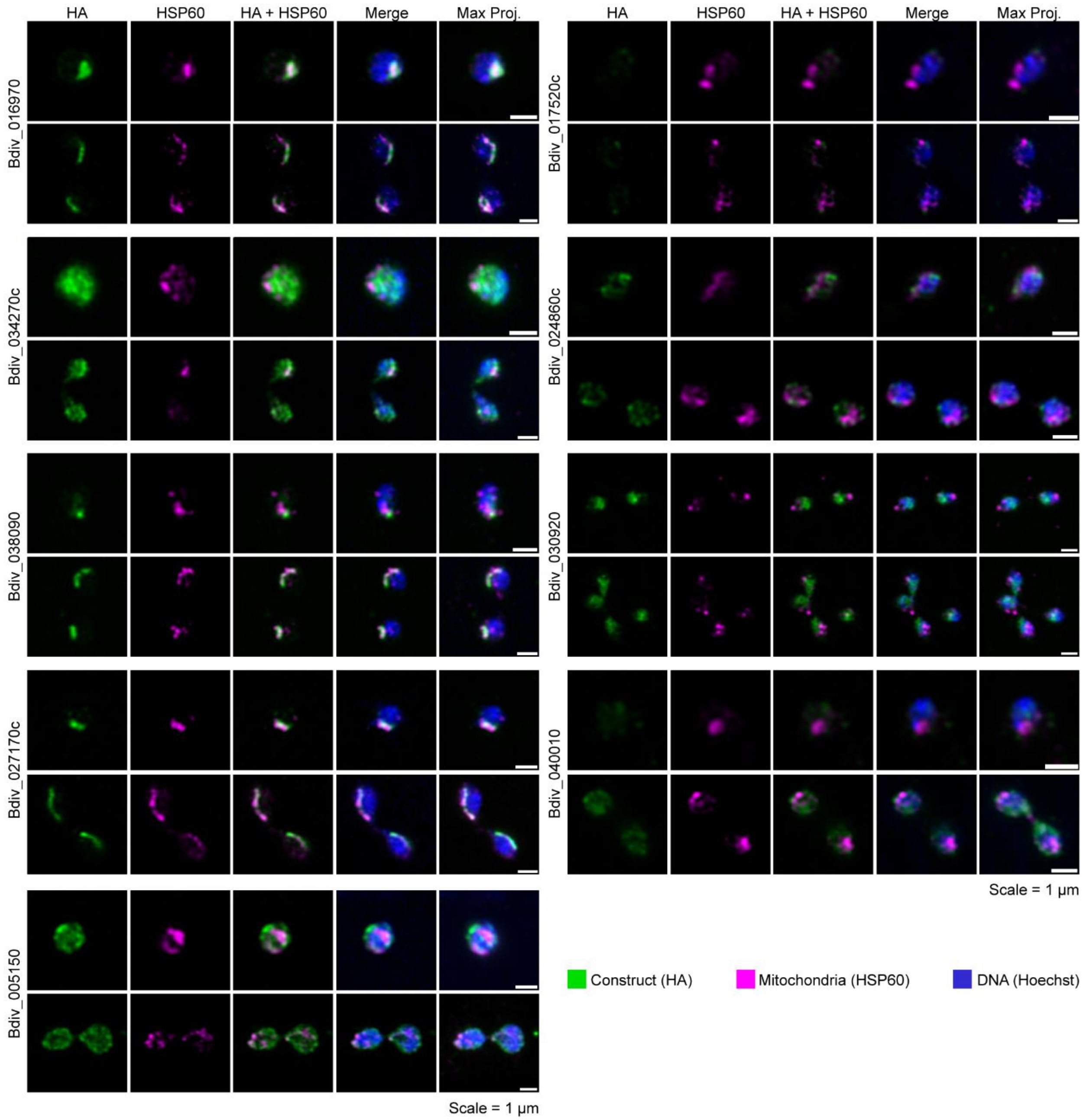

Figure S3. Additional immunofluorescence microscopy images for BdMitoCarta proteins and Gold+ proteins not in BdMitoCarta. Related to Figure 2.

Immunofluorescence microscopy analysis of construct localization in single and paired *B. divergens* merozoites. HSP60 was used as the mitochondrial marker. Scale 1 μm.

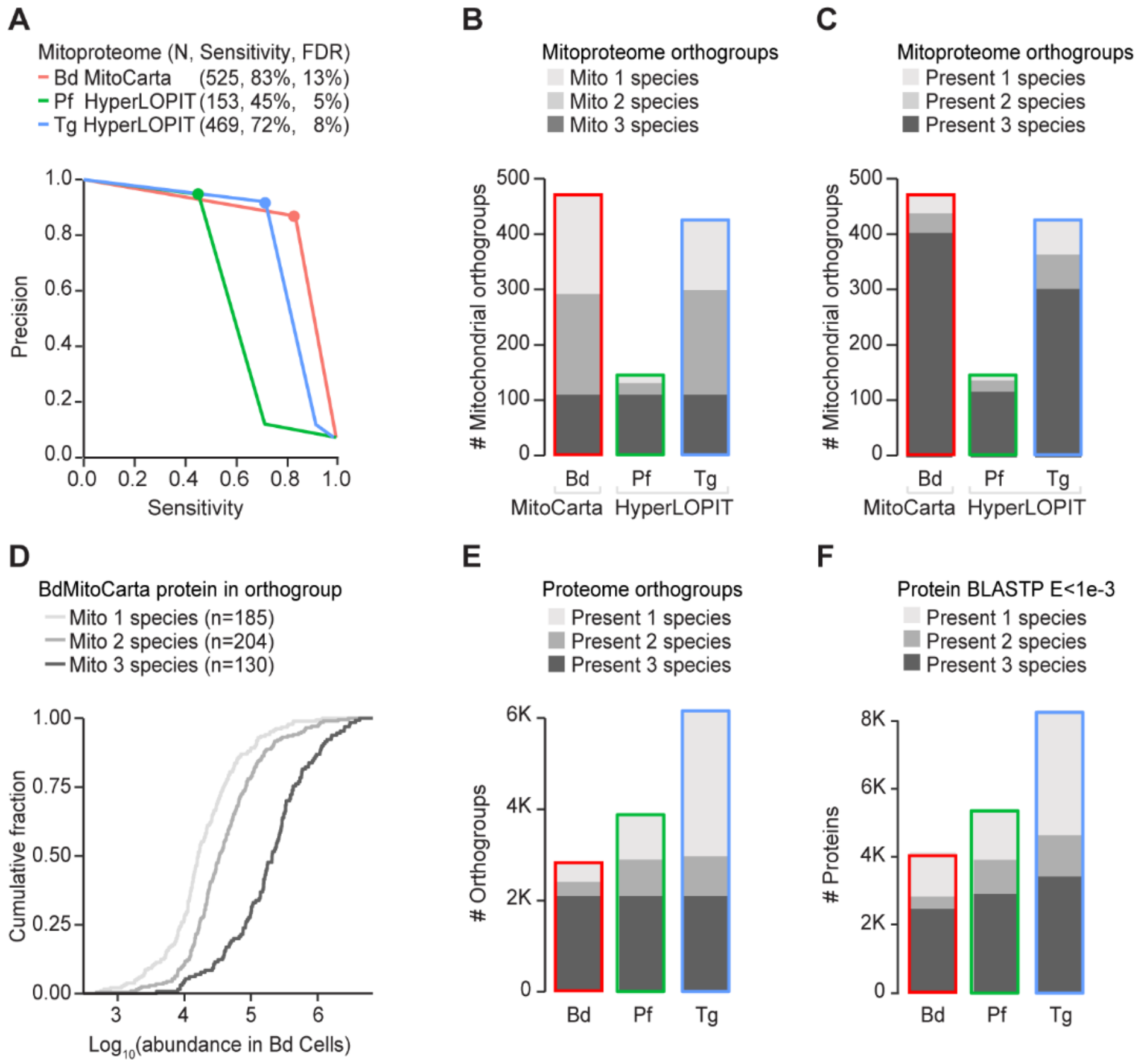

Figure S4. Comparison of BdMitoCarta to mitochondrial proteomes from hyperLOPIT in *P. falciparum* (Pf) and *T. gondii* (Tg). Related to Figure 3 and Data S4, S5.

(A) Sensitivity versus precision plots show estimated accuracy of mitochondrial inventories from *B. divergens* (MitoCarta, n=525) and published hyperLOPIT inventories in *P. falciparum* (Pf)[S3] (n=153) and *T. gondii* (Tg)[S2] (n=469) using the same benchmark methodology (see Methods). (B) Bar plots show comparison of mitoproteome inventories using orthogroups (see Chen et al, co-submitted), colors indicating if each orthogroup contains members in 1, 2 or all 3 mitoproteomes. (C) Bar plots show mitoproteome inventories colored by whether any orthogroup's members exist (mitochondrial or not) in the other species. (D) Empirical cumulative distribution function (eCDF) plot shows the abundance of BdMitoCarta proteins in whole cells, colored by whether each protein's orthogroup contains mitoproteome members in BdMitoCarta alone (light gray), also in Pf or Tg (gray), or all three mitoproteomes (dark gray). The proteins detected as mitochondrial in only *B. divergens* are typically lower abundance, potentially explaining why they might have been missed in other proteomics studies. (E) Bar plots show comparison of proteomes using orthogroups (see Chen et al, co-submitted), colors indicating if each orthogroup contain any members (mitochondrial or not) in 1, 2 or all 3 species. (F) Bar plots show comparison of proteomes using BLASTP homology instead of orthogroups, with colors indicating if each protein has any BLASTP hit (Expect < 1e-3) in the other species.

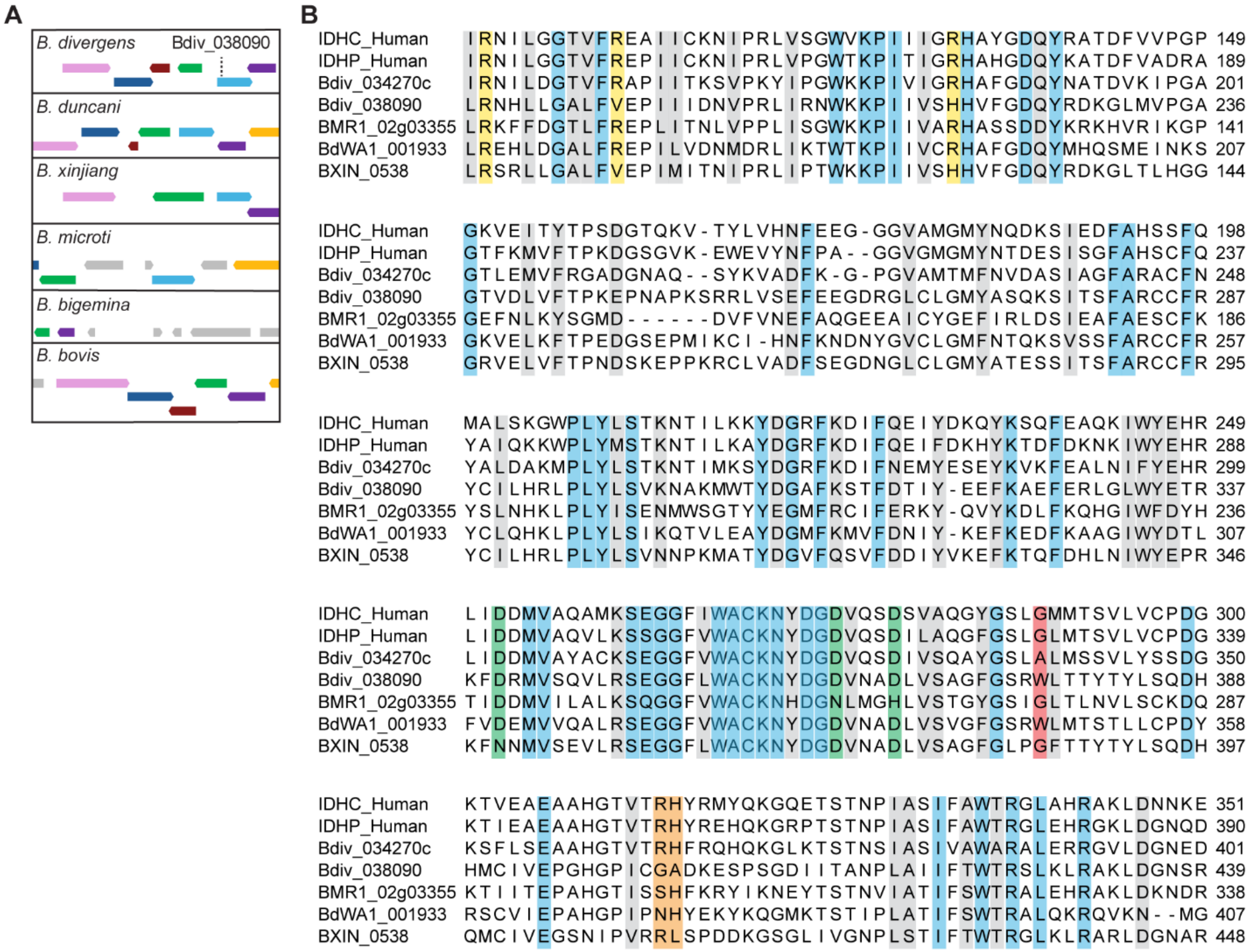

Figure S5. Synteny and alignments of *B. divergens* isocitrate dehydrogenase enzymes. Related to Figure 5.

(A) Synteny analysis of the Bdiv_038090 gene locus (reconstructed from PiroplasmaDB-68 synteny viewer, using the following reference genomes: bdiv1802A, BdunW12023, bspXinjiang, bmicRI, bbigBOND, bbovT2Bo). The order and orientation of genes surrounding the Bdiv_038090 locus is depicted for each *Babesia* species. Homologous genes are shaded using the same color. Genes without in-frame homologs are shaded in grey. (B) Sequence alignments of human and *Babesia* NADP$^+$-dependent isocitrate dehydrogenase enzymes (Clustal Omega).[S1] Colors denote residues discussed in the results: the arginine and histidine residues that coordinate the phosphate group of NADP$^+$ (Orange); the arginine residues that bind isocitrate (Yellow); the residues that bind magnesium (Green); the tryptophan residue of steric hinderance (Red). Grey shading indicates conservation of residues with similar properties and blue shading indicates a fully conserved residue.

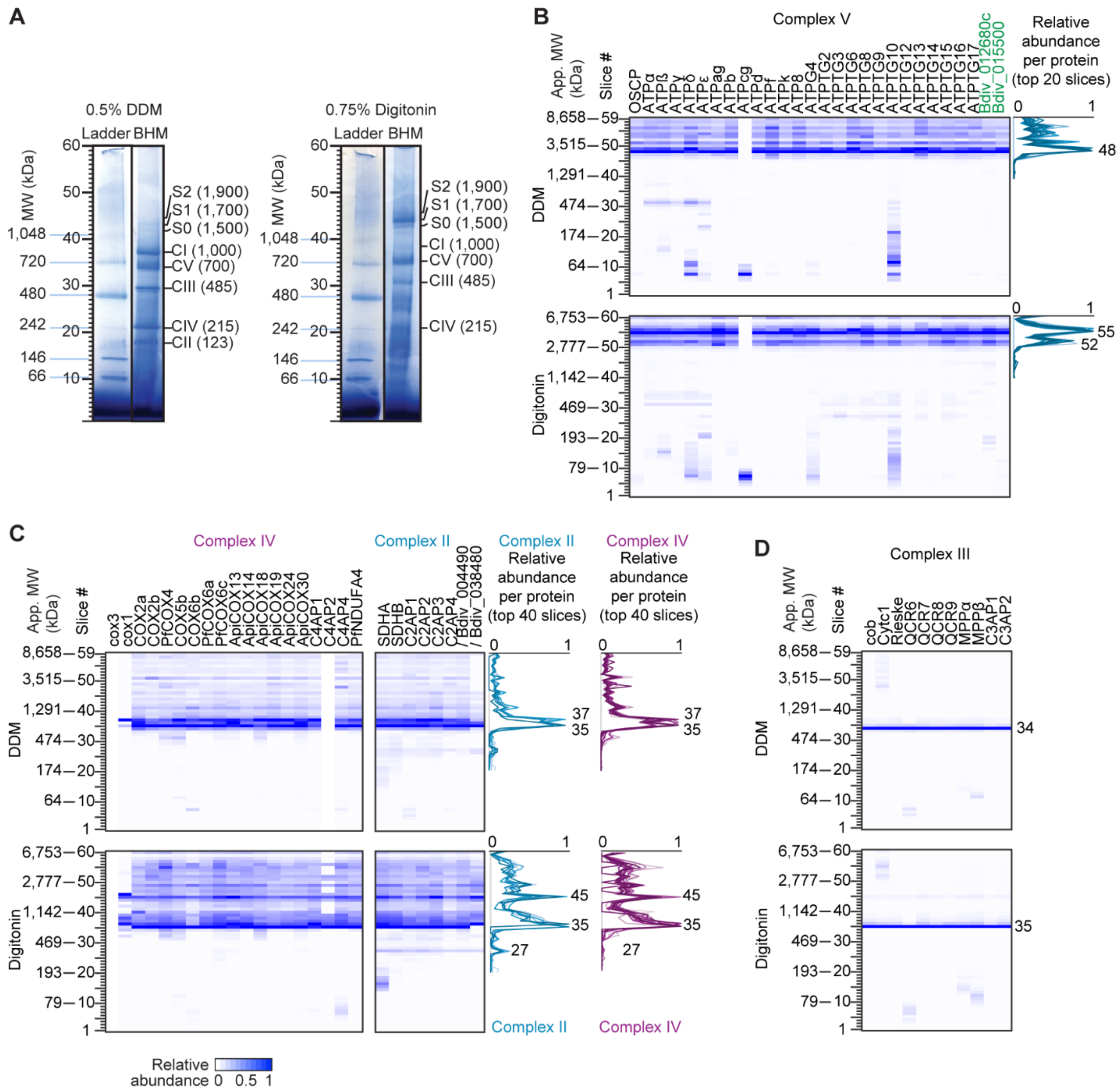

Figure S6. BN-PAGE migration of OXPHOS subunits and co-migrating proteins. Related to Figure 6 and Data S6.

(A) NativeMark unstained protein ladder (left) and bovine heart mitochondria (right) standards run with each sample preparation condition (0.5% DDM or 0.75% digitonin). The apparent molecular weight of each BN-PAGE gel slice was estimated using a linear model generated from the indicated OXPHOS complexes in the bovine heart mitochondria samples (Data S6D). (B) Heatmaps show relative abundances of BN-PAGE-MS results for known subunits of Complex V (blue) and co-migrating candidates (green), with names derived based on homology to known apicomplexan subunits. Line plots show the same relative abundance for the top 20 slices super-imposed with peak slices labeled. Heatmap relative abundance colors as in panel C. (C) Heatmaps show relative abundances of BN-PAGE-MS results for known subunits of Complex IV and Complex II. Line plots show the same relative abundance for the top 40 slices for Complex II (teal), Complex IV (magenta), and together. (D) Heatmaps show relative abundances of BN-PAGE-MS results for known subunits of Complex III.

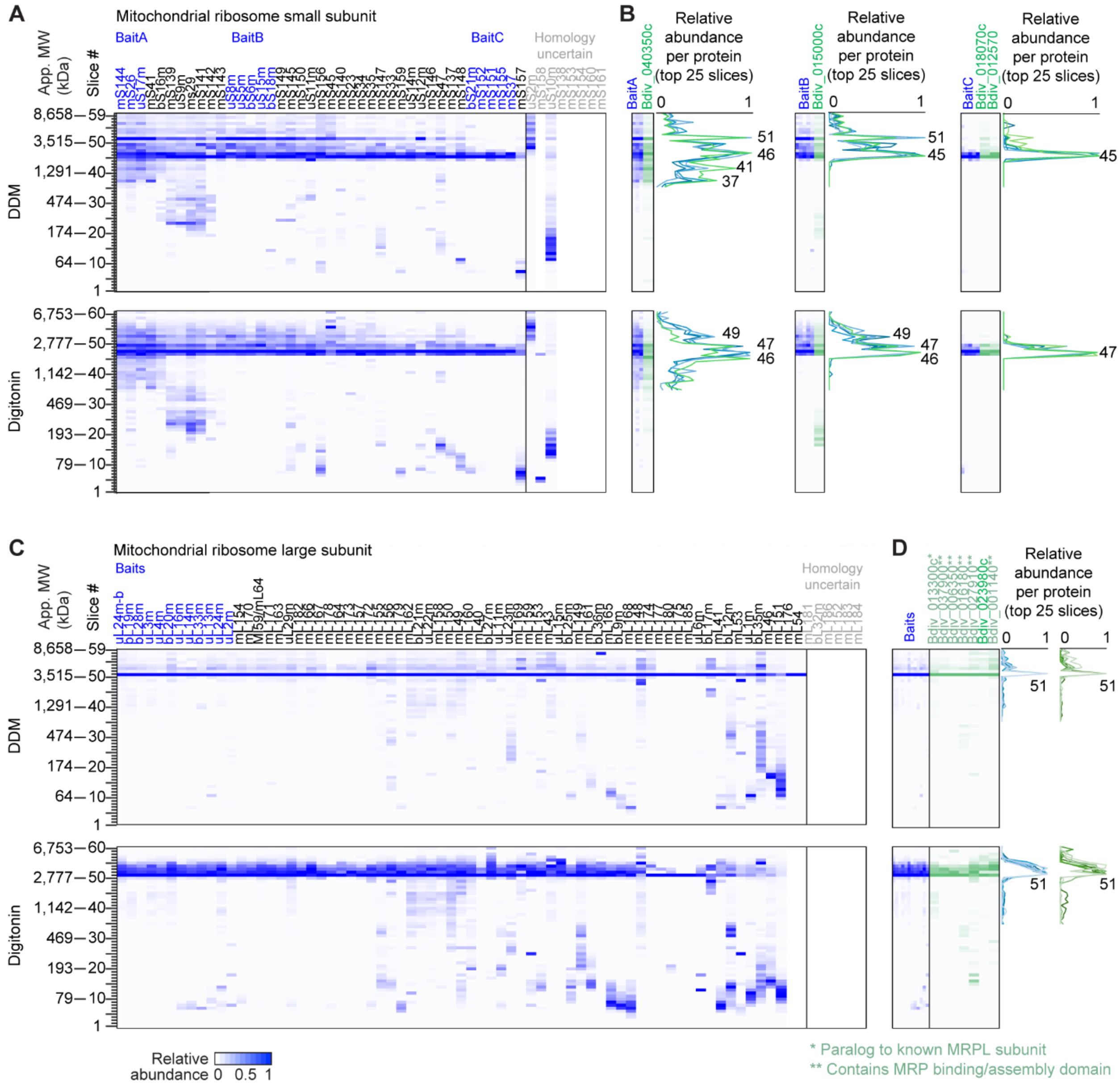

Figure S7. BN-PAGE migration of mitochondrial ribosome and co-migrating proteins. Related to Figure 6 and Data S6.

(A) Heatmaps show relative abundances of BN-PAGE-MS results for known structural subunits of the small subunit, based on homology to *T. gondii* (Tg) from Wang et al, 2024.[S4] Gray text indicates subunits unable to clearly resolve homology by BLASTP or orthogroups. Blue text indicates proteins used to define three bait sets (A, B, C) for co-migration analysis. (B) Heatmaps show relative abundances of BN-PAGE-MS results for three bait sets (blue) and co-migrating candidates (green). Line plots show the same relative abundance for the top 25 slices for baits (blue lines) and candidates (green lines). Numbers indicate peak slices. (C) Heatmaps show relative abundances of BN-PAGE-MS results for known structural subunits of the large subunit, based on homology to *T. gondii* (Tg) from Wang et al, 2024.[S4] Gray text indicates subunits unable to clearly resolve homology by BLASTP or orthogroups. Blue text indicates proteins used to define single bait set for co-migration analysis. (D) Heatmaps show relative abundances of BN-PAGE-MS results for the bait set in (C) (blue) and co-migrating candidates (green). Line plots show the same relative abundance for the top 25 slices for baits (blue lines) and candidates (green lines). Numbers indicate peak slices.

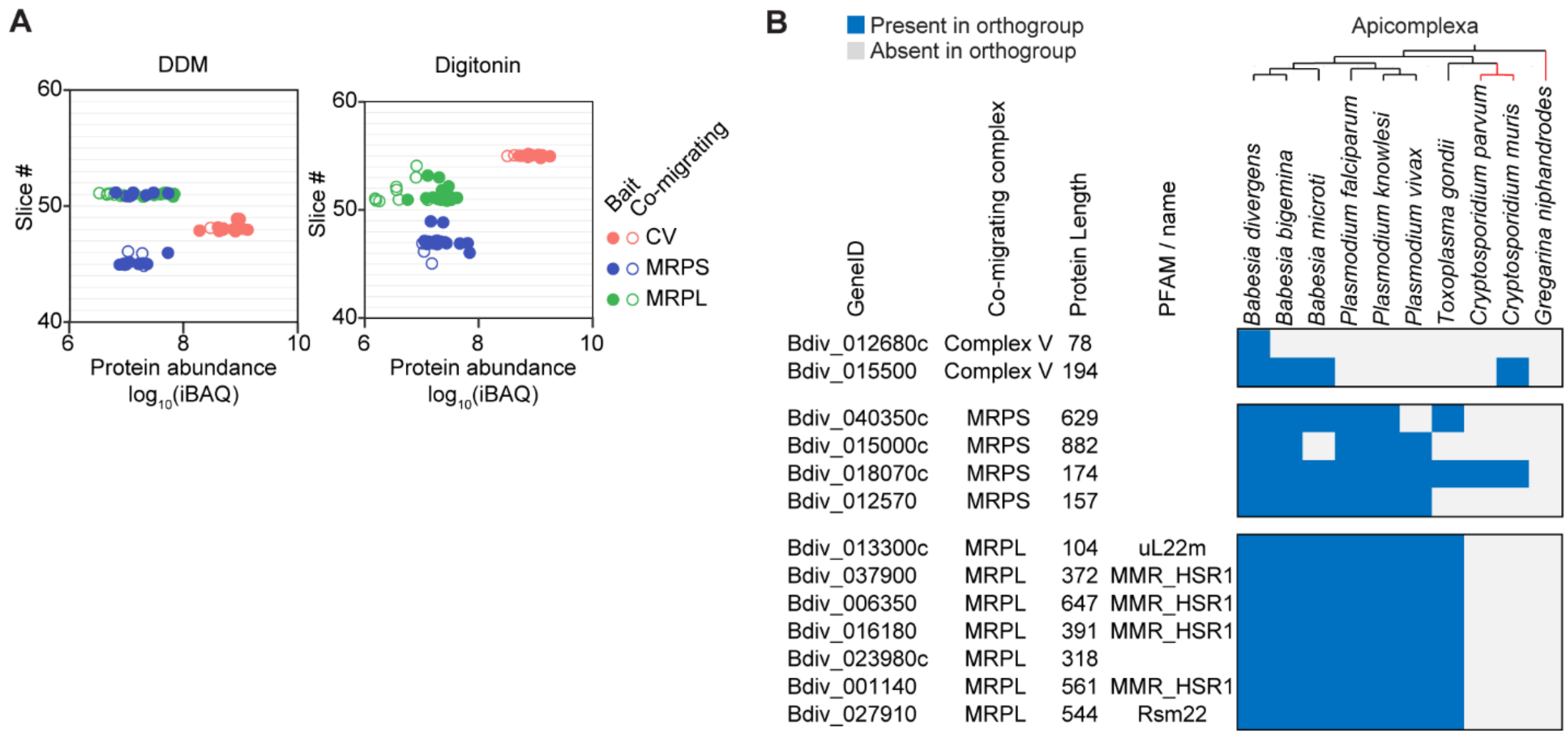

Figure S8. BN-PAGE proteins co-migrating with known complexes. Related to Figure 6 and Data S6. (A) Abundance of complex baits (filled circles) or co-migrating proteins (open circles) identified in each BN-PAGE slice based on iBAQ MS proteomics shows that baits and co-migrating candidates have similar abundance for CV and MRPS, whereas MRPL co-migration candidates show lower abundance than MRPL baits, consistent with assembly factors or binding proteins. For visualization, only slices with relative abundance ≥ 0.9 are displayed. (B) The co-migrating proteins are listed along with their known domains, and presence (blue) or absence (gray) in orthogroups from 12 selected apicomplexan species. In two cases (Bdiv_015500, Bdiv_018070c), presence of homologs in species lacking mtDNA (red lines) may suggest co-migration could be chance, since these species lack OXPHOS and mitochondrial ribosomes.

SUPPLEMENTAL REFERENCES

S1. Madeira, F., Madhusoodanan, N., Lee, J., Eusebi, A., Niewielska, A., Tivey, A.R.N., Lopez, R., and Butcher, S. (2024). The EMBL-EBI Job Dispatcher sequence analysis tools framework in 2024. Nucleic Acids Res *52*, W521-W525. 10.1093/nar/gkae241.

S2. Barylyuk, K., Koreny, L., Ke, H., Butterworth, S., Crook, O.M., Lassadi, I., Gupta, V., Tromer, E., Mourier, T., Stevens, T.J., et al. (2020). A Comprehensive Subcellular Atlas of the Toxoplasma Proteome via hyperLOPIT Provides Spatial Context for Protein Functions. Cell Host Microbe *28*, 752-766 e759. 10.1016/j.chom.2020.09.011.

S3. Chisholm, S.A., Flores, V., Kemp, A., Breckels, L.M., Koreny, L., Dos Santos Pacheco, N., Barylyuk, K., Kuroshchenkova, A., Lilley, K.S., Rayner, J.C., and Waller, R.F. (2026). The spatial proteome of the Plasmodium falciparum schizont illuminates the composition and evolutionary trajectories of its organelles. Nat Commun. 10.1038/s41467-026-73664-2.

S4. Wang, C., Kassem, S., Rocha, R.E.O., Sun, P., Nguyen, T.T., Kloehn, J., Liu, X., Brusini, L., Bonavoglia, A., Barua, S., et al. (2024). Apicomplexan mitoribosome from highly fragmented rRNAs to a functional machine. Nat Commun *15*, 10689. 10.1038/s41467-024-55033-z.
